# Rethinking Biomedical Titanium Alloy Design: A Review of Challenges from Biological and Manufacturing Perspectives

**DOI:** 10.1002/adhm.202403129

**Published:** 2024-12-23

**Authors:** Daisy Rabbitt, Victor M. Villapún, Luke N. Carter, Kenny Man, Morgan Lowther, Paraic O'Kelly, Alexander J. Knowles, Alessandro Mottura, Yuanbo T. Tang, Lorenzo Luerti, Roger C. Reed, Sophie C. Cox

**Affiliations:** ^1^ School of Chemical Engineering University of Birmingham Birmingham B15 2TT UK; ^2^ Department of Oral and Maxillofacial Surgery & Special Dental Care University Medical Center Utrecht Utrecht 3508 GA The Netherlands; ^3^ Regenerative Medicine Center Utrecht University Medical Center Utrecht Utrecht 3584 CT The Netherlands; ^4^ Paihau‐Robinson Research Institute Victoria University of Wellington Wellington 5010 New Zealand; ^5^ Center for the Accelerated Maturation of Materials Department of Materials Science and Engineering The Ohio State University 1305 Kinnear Road Columbus OH 43212 USA; ^6^ School of Metallurgy and Materials University of Birmingham Birmingham B15 2TT UK; ^7^ Alloyed Ltd Unit 15, Oxford Industrial Park Yarnton OX5 1QU UK; ^8^ Department of Materials University of Oxford Parks Road Oxford OX1 3PJ UK

**Keywords:** alloy design, biomedical alloy, implants, titanium

## Abstract

Current biomedical titanium alloys have been repurposed from other industries, which has contributed to several biologically driven implant failure mechanisms. This review highlights the added value that may be gained by building an appreciation of implant biological responses at the onset of alloy design. Specifically, the fundamental mechanisms associated with immune response, angiogenesis, osseointegration and the potential threat of infection are discussed, including how elemental selection can modulate these pivotal systems. With a view to expedite inclusion of these interactions in alloy design criteria, methods to analyze these performance characteristics are also summarized. While machine learning techniques are being increasingly used to unearth complex relationships between alloying elements and material properties, much is still unknown about the correlation between composition and some bio‐related properties. To bridge this gap, high‐throughput methods are also reviewed to validate biological response along with cutting edge manufacturing approaches that may support rapid discovery. Taken together, this review encourages the alloy development community to rethink their approach to enable a new generation of biomedical implants intrinsically designed for a life in the body, including functionality to tackle biological challenges thereby offering improved patient outcomes.

## Graphical abstract

This review advocates a shift in the mindset of biomedical alloy design from the current limited binary model (biocompatible and mechanically stable) to an approach centered on critical responses to numerous biological systems (immune, vasculature, and new tissue) while also protecting against significant threat, such as infection. It is proposed that this paradigm shift would support the evolution of a new generation of titanium materials specifically designed for function within the human body.

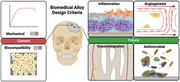

## 1 Introduction

Metallic devices are widely used to reconstruct hard tissues, equating to 70–80% of biomedical implants globally.^[^ ^1^ ^]^ Metals are commonly employed in joint replacement surgeries, including hip and knee, fixation and stabilization, such as spinal devices, and trauma‐based reconstructions, e.g. craniofacial plates.^[^ ^2^ ^]^ Generally, stainless steels, cobalt‐chromium (Co‐Cr) and titanium (Ti) based alloys are frequently used metallic biomaterials,^[^ ^1^ ^]^ with Ti and Ti‐based alloys being the most commonly used for implants due to their superior combination of mechanical, physical, and biological properties.^[^ ^2^, ^3^ ^]^ Given the high value and critical nature of these devices combined with a global orthopedic implant market size ($45.3Bn in 2021),^[^ ^4^ ^]^ it is surprising that only a handful of Ti alloy compositions are employed. A precise breakdown of alloys used by different implant manufacturers is difficult to obtain, however, ASTM standards for surgical implants exist for only seven different compositions.^[^ ^5^, ^6^, ^7^, ^8^, ^9^, ^10^, ^11^ ^]^ From these, most Ti implants are either grade 2 commercially pure titanium (cp‐Ti) or the grade 5 alloy Ti‐6Al‐4V. Despite the widespread use of these Ti‐based biomaterials, they were originally developed as structural materials within the aerospace industry.

Although these Ti‐based materials were not originally designed for biomedical functions, there are many advantages to their use. Cp‐Ti was recognized early as a broadly biocompatible material composed of hexagonal close‐packed (HCP) α phase at room temperature.^[^ ^12^ ^]^ In 1951, Leventhal used in vivo animal models to demonstrate that implanted titanium showed no notable adverse reactions and exhibited bone cell adhesion leading to its use as a surgical metal.^[^ ^13^ ^]^ It has high ductility (20% elongation) and relatively low strength (0.2% yield, 280 MPa) allowing it to be easily formed.^[^ ^12^ ^]^ However, this limits its use to low load‐bearing applications such as housings for active devices, dental implants, and cranio‐maxillofacial implants.^[^ ^14^ ^]^ Ti‐6Al‐4V is the most prevalent titanium alloy in use today for biomedical applications despite it being originally developed as an aerospace alloy.^[^ ^15^ ^]^ It has high specific strength (0.2% yield, 760 MPa) and good fatigue properties.^[^ ^12^ ^]^ This makes it an excellent candidate for weight‐bearing and cyclically loaded implants being widely used in hip and knee joints,^[^ ^14^ ^]^ where cp‐Ti is not appropriate. The presence of V stabilizes the body‐centered cubic (BCC) β phase, whilst Al stabilizes the α phase, leading to an α+β two‐phase microstructure at room temperature. Typically, it is processed to form a laminar α+β microstructure with controlled grain/lath size, which offers a good balance of mechanical properties.^[^ ^12^ ^]^ Variations in microstructure and mechanical properties of Ti‐6Al‐4V have also been shown to affect the corrosion resistance, in particular, higher hardness values equate to improved corrosion properties.^[^ ^3^ ^]^ An extra‐low interstitial (ELI, Grade 23) variant has tight controls on oxygen content and shows improved fracture toughness,^[^ ^16^ ^]^ with critical stress intensity values more than double that of the base alloy, further reducing the chance of fatigue failure. Arguably the success of high‐load bearing implants can be largely attributed to the performance of Ti‐6Al‐4V as an alloy. The UK saw a 37% increase in hip implants between 2008 and 2017 with more than 95% of these devices now expected to last more than ten years in service.^[^ ^17^ ^]^

Whilst these aerospace‐derived Ti‐based materials perform well in vivo, there remain some concerns around their use as a biomaterial.^[^ ^18^ ^]^ The growing uptake and increasing residency time of these implants have raised questions around the long‐term impact of alloying elements within the body. It has been shown that V is released from even well‐functioning Ti‐6Al‐4V implants and can be detected in blood, serum, and urine.^[^ ^19^ ^]^ Likewise, it is suggested that ionic vanadium may also be released into the body from the oxide layer^[^ ^20^ ^]^ and that metal ion release similar to that from a Ti‐6Al‐4V implant may inhibit normal cell differentiation.^[^ ^21^ ^]^ The precise mechanisms and severity of V toxicity, particularly in concentrations associated with Ti‐6Al‐4V release, remain an area of debate within the literature. Nevertheless, V has been associated with a variety of toxic effects including hematological, biochemical, neurobehavioral, and reproductive.^[^ ^22^, ^23^ ^]^ In vitro studies using rodent cells have specifically shown cytotoxic behavior and suggest that citrate, lactate, and chloride found in blood plasma may assist the release of vanadium ions whereby it bonds with human serum albumin enabling cell uptake.^[^ ^20^ ^]^

Concerns have also been raised regarding the release of aluminum either directly from the surface oxide layer or because of fretting.^[^ ^24^, ^25^ ^]^ Elevated Al levels have been detected in patients following hip implantation^[^ ^26^ ^]^ and shown to be released in ionic form from surface oxides when exposed to chemical species within the body.^[^ ^24^ ^]^ Some studies have suggested that Al is reintegrated locally into bone,^[^ ^27^, ^28^ ^]^ although the long‐term implications of this have not been reported. Overall, the impacts and concerns around V and Al highlight a need for Ti‐based biomedical alloys to be designed specifically for performance within the intended biological environment.^[^ ^29^ ^]^

New biomedical materials are essential in order to remove toxic elements to improve biocompatibility, better match the material properties (i.e., the elastic modulus) with those of bone, and enable the reduction in the size of some medical devices.^[^ ^30^ ^]^ However, the development process of biomaterials is lengthy, with new materials and processes frequently taking decades to be implemented.^[^ ^30^ ^]^ Historically, biomaterials have been repurposed from alternative industries, as such little research has been done on the interactions between these materials and the body.^[^ ^31^ ^]^ By the 1980s, a greater emphasis was placed on developing biomaterials with functional properties designed for their intended use by focussing on the bio interaction of the material with cells and the immune response.^[^ ^32^ ^]^ Newer alloys such as Ti‐Nb‐Ta‐Zr (TNTZ) and Ti‐Mo‐Zr‐Fe (TMZF) demonstrate a modulus closer to that of bone and only contain biocompatible elements, but have not yet been investigated over a wide range of compositions.^[^ ^33^ ^]^

Back‐tracking to list the traditional fundamental design requirements for an orthopedic implant essential indices include sufficient mechanical and structural support, whilst enabling integration with the damaged tissue and healing. As such the implant materials utilized should be biocompatible, non‐immunogenic, and, if required, facilitate osseointegration. The osseointegration of the material to bone allows a strong bond to form and therefore minimal movement occurs between the implant and tissue, aiding with the load‐bearing mechanisms.^[^ ^34^ ^]^ The biomaterial must therefore have good corrosion resistance, specifically in bodily fluid, and good wear resistance to ensure the patient suffers no adverse reactions from degradation products. The material must also exhibit high mechanical strength, toughness, fatigue resistance, low modulus, and low density.^[^ ^33^ ^]^

In recent years the field has begun to extend this traditional alloy design criteria list to include factors that may address biologically driven implant failure modes. For example, a mismatch of the Young's modulus (with common alloys used today being approximately three or four times stiffer than bone) may cause stress shielding resulting in a reduction in bone density and detrimental effects.^[^ ^35^, ^36^ ^]^ Premature failure of implants is often due to the initial inflammation and rejection. These responses can be triggered after initial implantation, or by wear and corrosion products, including debris or ionic species that may travel through the body and cause damage to cells or tissues, as well as in extreme cases, hypersensitivity, and cytotoxicity. Furthermore, any surgery can produce scar tissue and cause fibrous encapsulation, resulting in non‐bonding with surrounding tissue. Low fracture toughness or fatigue strength, can also lead to mechanical failure of the implant, often requiring immediate surgery.^[^ ^35^ ^]^ Another threat to orthopedic implants, and implants generally, is bacterial infections, which commonly require surgical action including the removal of the implant and debridement of any compromised tissue followed by lengthy antimicrobial therapies.^[^ ^37^, ^38^ ^]^

While there has been a movement towards offering solutions to these biologically driven implant issues little has filtered through into service. Currently, there are several dozen Ti‐alloys that are listed for having biomedical functions, and yet only seven have been standardized in ASTM for implant applications.^[^ ^5^, ^6^, ^7^, ^8^, ^9^, ^10^, ^11^, ^39^ ^]^ Thus, it is apparent that changes are needed in the approach to the development and adoption of new biomedical metals. The alloys currently used in the field, originally developed for aerospace applications, are not designed to combat biological threats. For example, considering the rise of antimicrobial resistance (AMR) has restricted infection treatment options, advanced alloys that could prevent or treat infections would be highly beneficial.^[^ ^40^ ^]^ Novel materials that are designed with these biomedical threats in mind could address the most common and costly implant failure mechanisms. With this in mind, we first offer a reflection on biomedical titanium alloy development, intended for permanent orthopedic devices, that is contextualized against progression within the aerospace industry. To support the progression of new alloy compositions an overview of key biological responses to implants is then presented. Finally, we review modeling and manufacturing techniques that along with high‐throughput biological validation methods would support the rapid progression of any new biomedical alloy compositions.

## 2 Biomedical Titanium Alloy Development: Lessons from Aerospace

Research efforts to remove the problematic alloying elements but maintain the α+β microstructure and mechanical properties of Ti‐6Al‐4V have resulted in numerous studies and suggested new alloy compositions. The general approach has been to substitute the phase‐stabilizing elements with those considered more “biocompatible”. βstabilizing elements such as niobium and tantalum^[^ ^14^ ^]^ are typically selected to replace vanadium. Both show excellent in vitro performance, similar to that of titanium, for cell proliferation.^[^ ^41^ ^]^ In vivo testing in a rodent model has demonstrated low inflammatory response and undetectable dissolution of both Nb and Ta in the surrounding tissue and surprisingly a significantly greater increase in new bone formation compared with titanium.^[^ ^42^ ^]^ Zirconium does not affect the relative stability of the α and β phases, but has been used as a substitute for aluminum to provide general solid solution strengthening of the α phase.^[^ ^14^ ^]^ Zirconium also shows good biocompatibility,^[^ ^41^ ^]^ without the broader medical concerns of aluminium.

Most notably, this approach of element substitution resulted in the development of Ti‐6Al‐7Nb (ASTM F1296^[^ ^5^ ^]^). Compared to Ti‐6Al‐4V, the two alloys perform similarly in yield strength (Ti‐6Al‐4V ELI: 795–875 MPa; Ti‐6Al‐7Nb: 880–950 MPa), ductility (Ti‐6Al‐4V ELI: 10–15%; Ti‐6Al‐7Nb: 8.1‐15%), and elastic modulus (Ti‐6Al‐4 V ELI: 101–110 GPa; Ti‐6Al‐7Nb: 114 GPa).^[^ ^43^ ^]^ Both also show similar corrosion^[^ ^44^ ^]^ and metal release profiles in relevant media;^[^ ^45^ ^]^ however, Ti‐6Al‐7Nb exhibits reduced toxicity demonstrated by cell attachment, proliferation, and viability.^[^ ^46^ ^]^ A number of alloys with the composition of Ti‐15Zr‐4Nb‐4Ta or similar have also been investigated and show comparable mechanical properties (Ti‐15Zr‐4Nb‐4Ta‐0.2Pd),^[^ ^43^ ^]^ low metal release,^[^ ^45^ ^]^ marginally reduced cytotoxicity, and improved in vivo tissue growth^[^ ^47^ ^]^ compared with Ti‐6Al‐4V. However, Ti‐6Al‐4V currently remains the gold standard for implant materials due to its proven clinical track record, good mechanical properties, and ease of manufacturing.

From a mechanical perspective, stiff Ti‐6Al‐4V joint prostheses are linked with “stress‐shielding” causing local bone loss and ultimately implant loosening. This effect is well documented and is typically attributed to the high stiffness of the implant with respect to the surrounding bone as a combination of material and design.^[^ ^48^, ^49^, ^50^, ^51^, ^52^, ^53^ ^]^ The compressive modulus of human cortical bone is in the range of 17.8 – 28 GPa, significantly below the 112 GPa of Ti‐6Al‐4V.^[^ ^54^ ^]^ Given the inherently lower modulus of the β phase,^[^ ^12^ ^]^ significant research activity has focussed on the development of a biomedical β (or near β) titanium alloy. Compositions containing the β‐stabilizing Mo, Nb, and Ta have typically been used to reduce both the β‐transus temperature and martensitic transformation temperature to produce a stable β material at room temperature.^[^ ^14^ ^]^ Development and evaluation of these alloys have been extensively reviewed,^[^ ^54^, ^55^, ^56^ ^]^ although special mention should be made to the Ti‐Nb‐Ta‐Zr alloy systems where moduli as low as 55 GPa has been achieved.^[^ ^54^ ^]^

**Figure 1:**
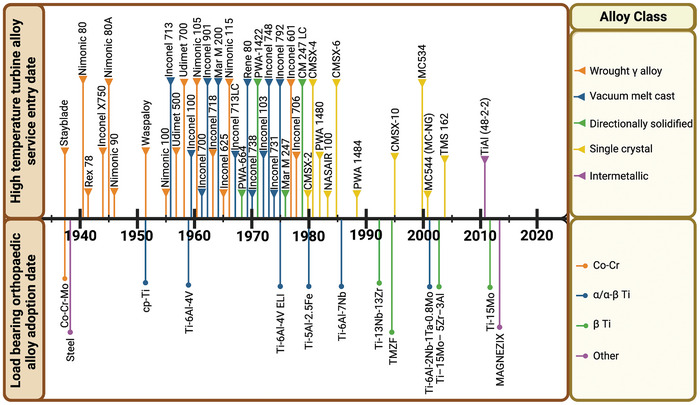
Timeline demonstrating the quantity of significant high‐temperature turbine alloys brought into service versus slower‐paced adoption of new alloys within load‐bearing orthopedic implants.^[^ ^59^, ^60^, ^61^, ^62^, ^63^, ^64^, ^65^, ^66^, ^67^, ^68^, ^69^, ^70^, ^71^, ^72^, ^73^, ^74^, ^75^, ^76^, ^77^, ^78^, ^79^ ^]^

Given that Ti‐6Al‐4V was originally developed as an aerospace alloy, another safety‐critical industry, it is interesting to compare the rate of new material uptake across these fields (**Figure** **1**). The aerospace industry has introduced new alloys at a much greater frequency than the biomedical industry, despite both requiring extensive testing and development before entering production. Since the development of Ti‐6Al‐4V in the 1950′s there have only been six further medical titanium alloy specifications registered with ASTM, by comparison approximately 33 new nickel superalloys for aerospace engines entered service between 1950 and the early 2000′s.^[^ ^57^ ^]^ It could be suggested that this demonstrates a lack of material innovation within the medical device field, however, this is not true. It has already been discussed that there exists extensive research to remove harmful elements and tackle stress‐shielding with orthopedic devices. As such the answer may lie in the drivers behind alloy development. The aerospace industry has benefited from two leading merit indices for high‐temperature turbine blade alloys: operating temperature and specific (yield and fatigue) strength. Both can be easily and precisely measured, and both are connected to tangible benefits. Increasing the operating temperature of materials (i.e., the temperature at which these alloys maintain their mechanical properties) directly allows for increased turbine entry temperature and improved engine performance.^[^ ^57^, ^58^ ^]^ Higher specific strength allows for mass reduction of parts whilst maintaining performance. Both provide increased efficiency at reduced operating costs and have driven innovation in aerospace alloy design and material processing. Alongside the continuous optimization of alloy composition for improved performance, new manufacturing processes were a significant factor in driving development and paradigm shifts in aerospace alloy design.

In the biomedical industry, there has been a tendency to judge alloys on two main criteria: biocompatibility and mechanical performance. As Ti‐6Al‐4V often meets the requirements for both, there is little perceived motivation for incremental improvements. In order to drive innovation, the metrics by which alloys are judged should be expanded to include functionalities not normally associated with the engineering properties of alloys. These may include reducing or eliminating bacterial attachment and growth, encouraging cell adhesion and integration, and promoting vascularization and material transport. In the same way that operating temperature and specific strength provide the aerospace industry incremental benefits with incremental improvements, better‐defined criteria could do the same for biomedical alloys. Advances in techniques for the rapid production of new alloys and the assays used to characterize them could serve an equivalent role in biomedical alloy development. Further metrics may be identified by engaging with stakeholders at all stages, from production to patient. New innovations can only occur by using this broader approach to challenge how medical alloys are discovered, validated, and translated to a clinical setting.

## 3 Biological Response

From the previous section, it is clear that the pool of available alloys for biomedical applications has been repurposed from alternative engineering fields, maintaining mechanical performance while adding biocompatibility as a critical criterion. A key concern for biomaterial scientists is the potential cytotoxic effects of implanted devices. Indeed, the first test needed to assess the viability of a novel device (ISO 10993) involves in vitro cytotoxicity analysis.^[^ ^37^, ^80^ ^]^ This focus on local and systemic toxicity is a critical step to guide the development of alloying systems for healthcare applications. Nevertheless, accounting for the potential toxicity on a fibroblastic or a fully committed osteoblastic cell line disregards key elements of the immune system, angiogenesis, and bone formation processes. With the main aim to provide more effective therapeutic solutions, novel biomaterials should be active participants in the modulation of wound healing, making it necessary to contemplate such effects from early development.

To ensure that an implant does not elicit cytotoxic responses care should be taken in selecting the metallic elements of a biomedical alloy. Obviously, non‐essential elements (e.g., Ga, Cd, Ag, Ge), that do not serve any biological function and could be toxic in small dosages should be removed. However, some essential ions serve fundamental roles in biological systems, but could risk metal homeostasis. Fe or Cu can be used as radical donors during the electron transfer chain, Zn or Mn are examples of metals required to form enzymes regulating and enabling various biochemical reactions, while Ca and Na are paramount to signaling and regulation.^[^ ^81^, ^82^, ^83^ ^]^ Metallic elements can also serve as targets to effectively halt and treat infection. The use of Cu and Zn in biomaterial design has gained traction as antimicrobial metals, however, these elements are harnessed by phagocytes to eliminate internalized pathogens.^[^ ^84^ ^]^ Similarly, some of these essential metals are required by pathogenic bacteria, causing an active competition for resources between the host and the infectious organism. The immune system can actively recruit these essential elements starving invading cells through a mechanism denominated as nutritional immunity.^[^ ^85^ ^]^ Variations in metal homeostasis as a consequence of increased essential or non‐essential metal uptake can catastrophically alter biological processes, leading to cancer or neurological, cardiovascular, and autoimmune diseases.^[^ ^81^ ^]^ Thus, it is necessary to understand the biological events that follow the implantation of a medical device and the potential effects that metals have on these processes.

This section provides such an insight, culminating in biological criteria to aid alloy discovery. Understanding the processes following implant placement enables the comprehension of the impact and functions of alloying elements. This understanding, in turn, facilitates the development of techniques for biological analysis. Biologically driven criteria should encompass the influence of inflammation on wound healing, angiogenesis, osseointegration, bacterial colonization, and biofilm formation. Additionally, new perspectives on each process are explored to introduce innovative assays and analyses that aid in advancing biological assessments.

### 3.1 Inflammatory Response

#### 3.1.1 Overview of Inflammatory Response to a Biomaterial

**Figure 2:**
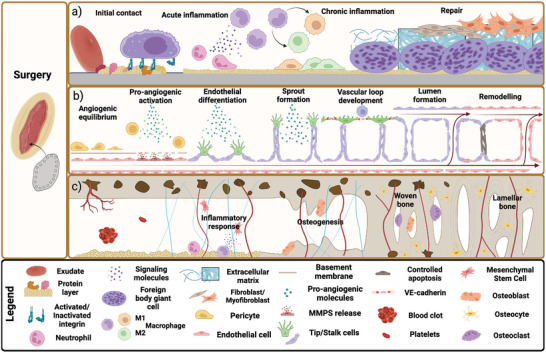
Depiction of the main stages taking place during a) wound healing, b) angiogenesis, and c) osseointegration.

The role of inflammation in wound healing is to contain, dilute, and eliminate any contaminants, effectively cleaning the wound site and enabling subsequent stages (**Figure** **2**a). Early inflammation occurs rapidly in a few days and is of short duration; exudation of fluids and plasma is followed by leukocyte adhesion. After an injury in a vascularized tissue like bone, exudation takes place; where fluid, proteins, and blood cells interact with the surface to provide a provisional matrix to support, and guide wound healing. Most cells make use of specific receptors (e.g., integrins) that recognize adhered molecules to offer focal points for adhesion and mechanosensing.^[^ ^86^ ^]^ During acute inflammation, neutrophils are the most predominant cells present in the injury site that proceed to recognize foreign material, guide the immune response, and phagocytose microorganisms.^[^ ^87^, ^88^ ^]^ This recognition will prompt neutrophils to release chemoattractants (interleukins IL1‐β and IL6 and TNF‐α), which will recruit circulating monocytes and promote their differentiation into their pro‐inflammatory phenotype.^[^ ^88^, ^89^ ^]^ The natural short life of neutrophils, 24 to 48 h, compared with that of macrophages, up to months, causes a shift in the cell environment where the latter becomes the main modulator of the subsequent inflammatory response and wound healing process.

Chronic inflammation, characterized by persistent inflammation, is the usual step in wound healing for implantable devices.^[^ ^80^, ^88^ ^]^ Monocytes, lymphocytes, and macrophages present at the biomaterial interface heavily characterize the transition from acute to chronic inflammation, which alongside neovascularization and proliferative tissue will form the foreign body reaction.^[^ ^90^, ^91^, ^92^, ^93^ ^]^ As part of other antigen‐presenting cells (APC), macrophages have a complex machinery of pattern recognition receptors, that makes them able to identify a myriad of macromolecules.^[^ ^89^, ^94^, ^95^ ^]^ In the case of an implantable device or particulates with sizes beyond the phagocytic capabilities of macrophages (100 µm in diameter) these cells generally agglomerate and differentiate into multinucleated macrophages, multinucleated giant cells (MnGC) or foreign body giant cells (FBGC), isolating the surrounding tissue.^[^ ^89^ ^]^ After this acute inflammation process, fibroblasts will be recruited and form the extracellular matrix (ECM), a physical scaffold and reservoir of modulators crucial for wound repair,^[^ ^96^, ^97^, ^98^, ^99^ ^]^ which is accompanied by neovascularization forming the granular tissue in the later stages of the wound healing process.^[^ ^87^ ^]^ The quantity of tissue lost during implantation of most devices limits the ability of parenchymal cells to fully restore the affected site, leading to a larger amount of granulation tissue and resulting in more fibrosis and scar tissue commonly observed after extensive surgery.^[^ ^100^ ^]^

#### 3.1.2 Role of Metallic Elements in Immune Modulation

Inflammation and wound healing are highly complex processes where the immune system plays a fundamental role throughout each stage. Uptake of metallic ions, even at low concentrations, can alter this fine balance by up‐ or downregulating immune responses. Neutrophils have been described as critical early respondents and orchestrators of the immune response, however, mercury, cadmium, or lead are only a few of the available metallic elements capable of impairing the phagocytic capabilities of these cells.^[^ ^101^, ^102^, ^103^ ^]^ On the other hand, proinflammatory interleukin IL‐1β secretion has been shown to be increased by Co, Cr, and Mo, resulting in damage to the lysosome of macrophages followed by reactive oxygen species (ROS) release.^[^ ^104^, ^105^ ^]^ ROS are one of the main antimicrobial weapons used by inflammatory cells, however, they are also highly genotoxic elements.^[^ ^87^ ^]^ Coupled with increased cell proliferation and an environment disassociated from the natural anticancer machinery of the host, this genotoxicity may lead to an increased presence of tumor cells and progression.^[^ ^87^ ^]^ In some cases, such as Cr, Ni, and Co, ROS release has been linked with modifications to signal transduction and gene expression in cells.^[^ ^106^, ^107^, ^108^ ^]^ On the other hand, V‐associated ROS generation in macrophages and epithelial cells has been demonstrated to activate transcription factor NFĸB, with subsequent release of proinflammatory cytokines and chemokines.^[^ ^109^, ^110^ ^]^

All these examples showcase the ability of metals to modify cell behavior and the inflammatory response of cells from the innate immune system. However, toxicological effects from metallic elements can also alter the response of the adaptive immune system. This part of the immune system is highly specialized to recognize and eliminate pathogenic cells and toxic molecules while retaining memories of these antigens to ensure long‐lasting protection. Some metallic elements can result in impaired functions of the adaptive immune response or, in the case of sensitizers, cause hypersensitivity after metal re‐exposure due to memory T‐cells releasing proinflammatory cytokines. For example, hypersensitization has been observed from Ni, Co, or Pd activation^[^ ^111^ ^]^ while Natural Killer (NK) cells have been shown to be impaired by Ni, Hg, Cd, Pb, and Zn.^[^ ^102^ ^]^ Enacting a long‐lasting inflammatory response or immune impairment is a clear indicator of the potential hazards posed by a novel alloy. Thus, an alloy's ability to maintain the regular functionality of the immune system and, ideally, modulate its response can be seen as biologically driven criteria.

#### 3.1.3 Methods for Immune Response Evaluation

The analysis of any potential irritation, sensitization, and/or immune toxicity effects of a medical device on the host is a requirement of ISO 10993. The gold standards proposed in these sections normally require the use of animal models, where the patch test or implantation at subcutaneous or intraperitoneal sites in small animals are two of the most commonly applied methods.^[^ ^112^ ^]^ Pre‐clinical in vivo models both in small and large animals may be required to fully understand the foreign body response of an implant. Their complexity, cost, and ethical considerations make such approaches impractical for early alloy development. Thus, in vitro assays are the main tool for the early evaluation of biologically driven success criteria of a prospective alloy. The early layer of protein adsorbed from exudates required for cell attachment and early signaling may be a simple test that could be used to validate a medical device. Increased protein adsorption alone does not necessarily suggest beneficial outcomes, with some reports showing that these materials can display pro‐inflammatory responses with an increased presence of leukocytes and macrophages followed by pro‐inflammatory molecule signaling.^[^ ^113^, ^114^, ^115^ ^]^ Consequently, in vitro cell work has become the preferred option with neutrophil‐based assays becoming more prominent in the literature^[^ ^116^, ^117^ ^]^ and some authors suggesting a broader use of lymphocyte proliferation test (LPT) as tools to evaluate adaptive immune system responses to implantable devices.^[^ ^118^ ^]^

#### 3.1.4 New Insights Into Biomaterial Driven Immunomodulation

More interesting from a biomaterial development perspective is the realization that macrophages are not a unique cell type, but multiple phenotypes capable of reversible differentiation with distinct roles in wound healing. These phenotypes can be generalized into two functionally different states, namely M1 and M2.^[^ ^119^, ^120^, ^121^ ^]^ M1 macrophages have been shown to release cytokines to induce rapid inflammatory and cytotoxic responses, while their M2 phenotype is characterized by the release of molecules enhancing angiogenesis, fibroblast proliferation and, in general, wound healing.^[^ ^113^, ^122^ ^]^ The ability to shift between phenotypes make macrophages an ideal marker for immune response and wound healing outcomes. Indeed, the capability of biomaterials to promote variations in M1/M2 differentiation has led to the application of a new paradigm for the development of novel biomaterials. With increasing reports on the possible correlation between M2 polarization and improved implant integration and wound remodeling,^[^ ^119^, ^123^, ^124^, ^125^, ^126^, ^127^, ^128^, ^129^ ^]^ it may be possible to generalize the use of macrophages as markers for novel alloy development. In the case of metallic alloys, these benefits can expand beyond a more reliable method for alloy discovery. By controlling macrophage polarization, the resulting fibrous layer that would effectively insulate an active device from the host tissue could be minimized, enabling the full potential of novel metallic alloys. Similarly, esterase secreted by macrophages could be reduced, limiting the adverse degradation of biomaterials^[^ ^89^, ^130^ ^]^ and the release of undesirable alloying elements. Still, it must be recognized that biomaterials should not completely inhibit M1 pro‐inflammatory responses, as this is still an important step for endogenous cell recruitment and activation during wound healing.

From this section, it is clear that wound healing is a complex phenomenon spanning numerous processes and cells working in tandem with the immune response acting as a critical interaction step. As beneficial as simple cytotoxic analysis may be for the early analysis of devices, it is difficult to elucidate if simple adhesion and proliferation of cells involved in the later stages of wound healing (e.g., fibroblasts or osteoblasts) can be used to estimate the downstream effectiveness of a biomaterial. While far from providing a detailed account of each and every interrelationship or to delve into the molecular biology that supports and enables this process,^[^ ^131^, ^132^ ^]^ a biomaterial scientist should now be aware of their importance and prospective methods to evaluate the immune response to novel alloys.

### 3.2 Angiogenesis

#### 3.2.1 Fundamental Aspects of the Angiogenic Process

Living tissue requires delivery of nutrients and oxygen, catabolic waste removal, and an interchange of molecular signals and cell mobility to dynamically respond to specific environmental cues, making vasculature critical for the correct function of biological processes.^[^ ^133^, ^134^ ^]^ Wound healing vasculature is achieved through branching and remodeling of already existing vessels and capillaries in a process known as angiogenesis. Blood vessels are multi‐layered structures with an inner shell of endothelial cells (ECs), followed by a layer of smooth muscle/mural cells separated by a basement membrane of interlinked proteins.^[^ ^135^, ^136^ ^]^ In normoxia, a balance is achieved between pro‐angiogenic (e.g., fibroblast growth factors, platelet‐derived growth factor, thrombin, etc) and anti‐ angiogenic (e.g., angionstatin, TIMP‐2) factors that suppress the formation of new capillaries. Hypoxia and inflammation shift this equilibrium to favor capillary growth^[^ ^134^, ^137^ ^]^ (Figure 2b). More specifically, the upregulation of vascular endothelial growth factor (VEGF) leads to pericyte decoupling and stimulation of metalloproteinases (MMPs) production, exposing the underlying basement membrane and proceeding to its cleavage. As this process continues, further angiogenesis factors entrapped in the membrane will be liberated promoting differentiation of the exposed ECs into two transient phenotypes regulated by the Notch pathway, DLL4 ligands, and VEGF receptors 1, 2, and 3.^[^ ^138^, ^139^, ^140^ ^]^

During the first stages of VEGF interaction, exposed ECs will develop filopodia and sprouts turning into tip cells, capable of sensing and following angiogenic clues to promote new vessel formation.^[^ ^138^ ^]^ Increased tip cell population results in a negative control loop, limiting tip cell formation and promoting stalk cell differentiation. As a consequence, sprouts of stalk cells will be developed throughout the exposed regions of existing vessels spearheaded by tip cells following a gradient of angiogenic factors.^[^ ^134^, ^138^ ^]^ Further development causes anastomosis or the creation of connections between neighboring tip cells in a process facilitated by macrophages,^[^ ^138^, ^141^ ^]^ following cell reorganization and lumen formation.^[^ ^142^, ^143^ ^]^ In the next steps, differentiation of mural cells, pericyte recruitment, and extracellular matrix deposition will confer stability to the network that coupled with blood flow will activate stress‐responsive genes (e.g., KLF2), promoting quiescence of ECs dilation of the tubules, perfusion and prevent clot formation.^[^ ^134^, ^138^, ^144^ ^]^ Finally, selected apoptosis takes place in vessels without hemodynamic stimulation promoting the remodeling of large sections of the newly formed capillaries^[^ ^137^ ^]^ (Figure 2b).

#### 3.2.2 Influence of Metallic Elements on Angiogenesis

Based on the critical role that angiogenesis plays during the wound healing process, metals capable of its modulation could provide considerable benefits to implantable devices. In this regard, Co and Cu have been the most studied hypoxia‐mimicking materials due to their ability to imitate the lower oxygen conditions defining early pro‐angiogenic activation. These elements have been shown to stabilize hypoxia‐inducible factor (HIF)−1.^[^ ^145^, ^146^ ^]^ The resulting increase in HIF‐1 causes an upregulation of VEGF and other pro‐angiogenic factors enhancing vasculature formation and blood supply for these elements both in vitro and in vivo.^[^ ^147^, ^148^, ^149^, ^150^ ^]^ Similarly, Zn has been shown to promote angiogenesis through HIF‐1 stabilization, though their beneficial properties can also be ascribed to increased levels of VEGF resulting from macrophage polarization.^[^ ^147^, ^151^ ^]^ This phenomenon is also observed in Cu, Sr, Si, or Eu.^[^ ^151^, ^152^, ^153^ ^]^ While the mechanisms behind this effect on macrophages—and in some instances endothelial cells—is still not fully understood, some authors have stated the inherent ability of metallic elements to act as ROS promoters and quenchers. In this regard, studies on Ce have revealed its potential as a radical regulator by modifying oxidation states between Ce(IV) and Ce(III) in physiological conditions, exerting a protection effect against oxidation stresses in endothelial and progenitor cells leading to improved angiogenesis.^[^ ^154^, ^155^ ^]^ Other pro‐angiogenic elements display similar indirect effects by targeting proliferation and cell migration. For instance, Mn can direct cell movement by modifying cAMP activity, cytoskeleton‐related genes, and actin dynamics,^[^ ^156^, ^157^ ^]^ and is well known to influence focal adhesion and actin filament formation.^[^ ^158^, ^159^ ^]^ It can also act as an insulin‐mimetic compound leading to VEGF upregulation and subsequent increase in vascularization and wound healing.^[^ ^160^ ^]^ The established behavior of these elements and others (e.g., boron, vanadium, or lanthanum),^[^ ^147^, ^158^, ^161^ ^]^ reveal the potential use of metallic elements to provide active functionalization for improved angiogenesis. It is, however, of critical importance to ensure dosing and form do not compromise other biological processes. Elevated Co or Cu levels may result in reduced mineralization^[^ ^158^, ^162^ ^]^ while the pro‐angiogenic effects of Tb, Au, Ag, or W have been mostly demonstrated as nanoparticulates,^[^ ^163^ ^]^ limiting their translation to metal alloys. Testing the potential therapeutic effects of novel multicomponent alloys and fine‐tuning the delivery of active elements through relevant assays is essential to ensure efficacy and safety.

#### 3.2.3 Conventional Strategies for the Analysis of Angiogenic Responses

Given the central role that angiogenesis has on inflammation, mineralization, and wound healing coupled with the potential benefits of materials capable of guiding this process, it is surprising that limited angiogenic validation assays can be found in the early development of metal implantable devices. Effective vascularization is indispensable in basic cell function with cells requiring access to a vascular network within 200 to 400 µm of the cell.^[^ ^164^, ^165^ ^]^ Available reports have shown that the failure of medical devices can often be ascribed to a poorly interconnected vascular network.^[^ ^166^ ^]^ From an alloy development perspective, it is important to start analyzing their prospective influence during the early stages of development for which simple, inexpensive, and scalable in vitro methods can be used. These methods normally make use of endothelial cells to study variations in proliferation, differentiation, migration, and reorganization.^[^ ^167^, ^168^, ^169^ ^]^ The simplest modes of analysis involve the direct counting of cells in contact with a specific material, albeit other indirect methods based on metabolic assays (e.g., MTT) or more specific cell cycle staining protocols (e.g., BrdU and propidium iodide) can be employed. More specific methods can assess induced variations in motility or sprout and lumen formation, migration, and tubulogenesis assays, respectively. In the former, techniques such as the scratch assay or Boyden chamber assay are capable of studying the ability of endothelial cells to bridge a gap created in a confluent layer or to pass through a permeable filter separating a chemoattractant and the desired cell line.^[^ ^168^ ^]^ In contrast, tube formation assays quantify the number, density, and branching of newly formed sprouts developed by endothelial cells embedded or in contact with a soft hydrogel consisting of collagen, fibrin, or, more generally, Matrigel.^[^ ^167^ ^]^ Using a combination of these in vitro techniques, it is possible to emulate activation, growth, and sprout/tube formation to understand the potential effects of new treatments in angiogenesis. However, these techniques are not capable of reproducing the 3D architecture, microenvironment, and cross‐talk between numerous cell types, making animal models necessary in the safety and validation stages.

To provide reliable platforms to enable the study of angiogenesis and related diseases, there exists a plethora of animal models available for testing novel molecules and biomaterials. From these, the chick chorioallantoic membrane (CAM) assay quantifies angiogenesis through the number and density of vessels formed in a chicken embryo subjected to the tested substance either through a window perforated in the shell, in ovo, or by placing the growing embryo in a culturing plate, *ex ovo*. Its relative simplicity, possibility of scaling for high throughput analysis, capability of multiple measurements, and analysis of different dosages have made it one of the most used in vivo angiogenesis models.^[^ ^170^, ^171^ ^]^ The lack of an immune system, crucial for wound healing,^[^ ^172^, ^173^ ^]^ limits its application. Alternative methods commonly used include the dorsal air socket model, subcutaneous implantation or plug assays, and the chamber tests, where either an air cavity is formed or a chamber with a glass slide implanted to deliver treatment, directly or injected in a suitable medium (e.g., Matrigel). To measure variations in angiogenesis, some of these methods require the retrieval and histological evaluation of the implanted material, such as in the case of a Matrigel plug, while chambers enable real‐time assessment of tubulogenesis.^[^ ^167^ ^]^ While all these techniques are capable of better emulating the real environment and process of angiogenesis, their complexity, technical expertise, cost, and ethical implications make them difficult to use for high throughput methods. As a result, the analysis of angiogenesis in zebrafish has become popular as the small size of these animals, rapid life cycles and easily measurable vessel formation make possible the study of multiple subjects and treatments in situ over time.^[^ ^167^, ^168^ ^]^ However, the relevance to the results obtained when compared to human responses, as well as the difficulty in differentiating neovascularization from angiogenesis, still present issues for its broad spread use in biomaterial discovery. As a middle step between in vitro and in vivo, analysis of sprouting and tube formation from rat or pig aorta explants into collagen or other hydrogel matrix can be a valid alternative. There is, however, no consensus in the research community for a gold‐standard angiogenesis assay, requiring several methods to be performed to properly assess efficacy and safety.^[^ ^168^ ^]^

#### 3.2.4 Future Perspective on Assessment of Angiogenesis

For the aforementioned angiogenic assessments to be used in alloy development there are a number of important considerations. First, most of these methods have been optimized for the analysis of deliverable drugs in liquid form or suspended in a hydrogel, requiring modifications to enable their use in alloy discovery. A common approach includes the exploitation of ISO 10993 to extract leachables that can then be processed and analyzed.^[^ ^174^, ^175^, ^176^ ^]^ Other alternatives include the assessment of angiogenesis in common in vivo bone assays such as the calvaria and long bone models, or variations in CAM, (e.g., the chick limb organ culture), which allow the implantation of bulk materials.^[^ ^172^ ^]^ Thus, it is especially important for the future of biomedical alloys to consider the available techniques for the selection of a proper array of tests and, if necessary, consider their modification to ensure no safety issues arise from direct implantation. When numerous alloys are to be considered, there are still limited techniques enabling the high throughput analysis of angiogenesis with spheroids, organoids, 3D cultures, scaffolds, 3D printing, and microfluidic‐based angiogenesis scalable platforms.^[^ ^177^ ^]^ For instance, Kim et al.^[^ ^178^ ^]^ and Duinen et al.^[^ ^179^ ^]^ have described microfluidic chambers capable of studying the tubulogenesis of endothelial cells for the screening of angiogenic molecules with the latter adapting their system to a modified 384 well‐plate. These novel models are still under development, requiring further validation with in vivo data.^[^ ^166^ ^]^ Consequently, material scientists focused on manufacturing novel metallic alloys capable of guiding biological processes should aim to analyze angiogenesis from an early stage while recognizing the need for the adaption of current methods for bulk samples. Thereby highlighting a demand for new reliable and scalable angiogenesis assays in vitro.

### 3.3 Osseointegration

Bone is a complex agglomeration of collagen, multi‐substituted hydroxyapatite, and water with proteins, lipids, and polysaccharides at low quantities,^[^ ^180^ ^]^ which can be further subdivided as either cancellous or cortical bone due to their structure and mechanical properties.^[^ ^181^ ^]^ While the process of bone formation can differ between areas, such as intramembrane or endochondral ossification in flat or long bones respectively, from an implantable device perspective the process of osseointegration better describes these interactions. Osseointegration is defined as a time‐dependent process of tissue healing where rigid fixation of foreign materials to bone tissue is achieved and maintained during biomechanical loading.^[^ ^182^ ^]^ For many implants it represents the ideal scenario, providing the current best mechanical fixation between the host's skeletal system and device.

#### 3.3.1 Fundamentals of Osseointegration

The process of osseointegration is typically described in five stages (Figure 2c) sharing the first two with the wound healing process. The initial trauma to the bone matrix, results in the release of soluble proteins,^[^ ^183^ ^]^ providing a layer to facilitate the adhesion of cells to implant surfaces.^[^ ^184^ ^]^ At the same time, a provision matrix is created on the implant surface through the formation of a fibrin clot, containing a plethora of bioactive signaling factors including mitogens, cytokines, chemoattractants, and growth factors.^[^ ^185^ ^]^ This is followed by the early inflammatory response with neutrophil recruitment and subsequent monocyte and macrophage infiltration into the peri‐implant gap. These cells are involved in the removal of debris and necrotic cells but, more importantly for osseointegration, secrete a range of growth factors and cytokines that stimulate the recruitment, proliferation, and differentiation of mesenchymal stem/stromal cells (MSCs).^[^ ^186^ ^]^ MSCs are a subpopulation of multipotent cells that reside within the bone marrow and are responsible for giving rise to all cells of the mesoderm lineage.^[^ ^187^ ^]^ Crucially, these MSCs differentiate into cells responsible for modulating bone formation; osteoblasts, osteocytes, and osteoclasts.^[^ ^188^ ^]^

During the osteogenesis process, the gap between the implant and the native bone gives rise to two clearly differentiated bone formation processes. Contact osteogenesis takes place on the surface of the implant through the differentiation of osteoprogenitors to osteoblasts,^[^ ^189^ ^]^ which produce the osteoid. This woven matrix consists mainly of collagen type I and non‐collagenous proteins (e.g., osteonectin, osteocalcin, and osteopontin) that are then mineralized through the formation of hydroxyapatite crystals.^[^ ^190^, ^191^ ^]^ On the other hand, distant osteogenesis will take place at the host bone tissue by forming a fibrin bridge that will facilitate bone matrix migration.^[^ ^189^ ^]^ It has been reported that these two osteogenic routes interact synergistically to promote osteogenesis and gap bridging, forming a characteristic irregular trabecular pattern with randomly oriented collagen fibers and a high number of osteocytes.^[^ ^192^ ^]^ After the filling of the bone‐implant gap via the formation of woven bone, this tissue is subsequently remodeled by terminally differentiated multinucleated cells or osteoclasts, which is dictated by the direction of mechanical loading applied to the tissue.^[^ ^193^, ^194^ ^]^ In this process, osteoclasts remove microcracks and prepare the surface with micro/nanotopographies, and biochemical cues to guide osteoblasts mineral deposition.^[^ ^195^ ^]^ In contrast, osteoblasts will regulate osteoclast differentiation through the receptor activator of NF‐κB ligand (RANKL) and osteoprotegerin (OPG) signaling pathway, resulting in a critical balance for appropriate bone remodeling. Differentiated osteoclasts affix to the bone surface though integrin‐osteopontin interactions,^[^ ^196^ ^]^ secreting hydrochloric acid to demineralize the bone matrix and cathepsin K to digest the collagen matrix. Osteoblast precursors are able to recognize the topography of the digested bone and determine the volume of bone required to fill the defect gradually replacing the woven bone into lamellar bone.^[^ ^197^ ^]^ Osteoblasts then terminally differentiate into osteocytes that are embedded within the mineralized matrix of bone^[^ ^198^ ^]^ and capable of responding to external stimuli through mechanosensing.^[^ ^199^ ^]^ Osteocytes also secrete a range of signaling molecules that regulate the activity of osteoblasts and osteoclasts^[^ ^200^ ^]^ so that, after 12 weeks, the implant surface is completely adjoined by mature lamellar bone.

#### 3.3.2 Potential Effects of Metals in Osseointegration

In designing a novel alloy, one should consider the effect of each constituent, their interactions, and variations in microstructure realized during material processing that may impact any step in osseointegration. Each element could alter MSCs recruitment and their ability to proliferate or differentiate, influence extracellular matrix production, mineral nucleation, and crystal growth, vary osteoblast/osteoclast behavior and subsequent bone formation and remodeling, or even modify homeostasis of elements critical for bone formation. These provide clear objectives to guide therapeutics, however, they also present numerous targets for toxic elements. For example, Cd has previously been shown to negatively impact the immune response, with recent reports extending its toxicological impact to bone formation. Specifically, it has been shown to limit the expression of osteoblast differentiation markers (e.g., Runx2, osteocalcin) and bone matrix development (e.g., type I collagen), enhance osteoclastogenesis via RANKL expression and limit alkaline phosphatase (ALP) production, critical in the early stages of mineralization.^[^ ^201^, ^202^ ^]^ ALP has also been shown to be negatively impacted by nickel and chromium, alongside increased apoptosis of osteocytes in the former and reduced viability of both osteoblasts and osteoclasts in the latter.^[^ ^203^, ^204^, ^205^, ^206^ ^]^ Al has been proposed to affect bone homeostasis by decreasing levels of Ca, Mg, and P, whilst reports have also displayed its negative impact on several osteoblast differentiation pathways, bone formation markers, and osteoblast viability.^[^ ^207^, ^208^ ^]^ Similarly, Fe can limit the nucleation of nodule minerals and alter hydroxyapatite crystallinity, alongside potential negative effects across all aforementioned processes.^[^ ^209^, ^210^ ^]^

While our understanding of the role of specific metals and metalloids in these processes is still increasing, both in vitro and in vivo works have provided specific candidates that are currently being explored to guide bone healing. As two of the main components of bone, most of the work available in the literature has focused on the role of calcium and phosphate in providing biocompatibility and rapid osseointegration of implants. Their influence on cell behavior and ability to modify bone formation throughout all stages is well documented,^[^ ^211^ ^]^ however, their physical properties and the need to obtain specific phosphate formulations have primarily led to applications in the form of coatings with limited work available on the Ti‐Ca system.^[^ ^212^ ^]^ Advances over the last decade have shown the potential benefits of alternative elements, with most work focused on Zn, Sr, Mg, or B. The first of these metals has gained traction in biomaterial development due to its antimicrobial properties, however, the ability of Zn to regulate ALP levels in a dose dependant manner has made it of clear interest for bone regeneration.^[^ ^213^ ^]^ More importantly, Zn has showcased an ability to enhance osteoblast differentiation while limiting osteoclastogenesis,^[^ ^214^ ^]^ resulting in an effective double mode of action to promote osseointegration. A similar effect has been displayed by Mg,^[^ ^215^, ^216^ ^]^ albeit its rapid degradation in physiological environments requires specific considerations to ensure the mechanical stability of the implantable device. The biodegradable nature of Mg has thus been widely investigated in combination with Ti to construct smart implants with “semi‐degradable” properties.^[^ ^217^, ^218^, ^219^, ^220^, ^221^, ^222^, ^223^, ^224^ ^]^ Sr, a homologous element to Ca, has been shown to influence ALP and bone sialic acid glycoprotein production, regulate the receptor activator of NF‐kB ligand (RANK/RANKL) pathway, and inhibit mRNA expression in osteoclasts, making it one of the most popular elements in bone regeneration.^[^ ^215^, ^225^ ^]^ More recently, studies have revealed the ability of boron to influence bone formation genes, however, the mixed pool of manuscripts demonstrating its efficacy may require further work to establish its benefits in biomedical alloys.^[^ ^161^, ^211^ ^]^ Besides these elements, benefits have been shown in the use of Mn, Nb, and V in ECM production, osteoblast activity, and osteochondral development, respectively, which alongside Ga, Li, Si, or F may be elements of consideration for the design of novel alloying systems.^[^ ^145^, ^161^ ^]^

#### 3.3.3 Available Assays for Evaluation of Osseointegration

To fully exploit the benefits of all the aforementioned elements, it is necessary to ensure both the safety and functionality of the novel alloy. These properties have been historically assessed through a combination of early proof of concept development using in vitro 2D cultures and animal models to test a reduced subset of potential candidates for future clinical evaluation.^[^ ^226^, ^227^ ^]^ The need for a material to be safe locally and systemically translates into an array of undesirable outcomes (e.g., toxicity, thrombogenesis, carcinogenicity, mutagenesis, or immunologic response) dependant on the interrelations between several cell types. In the case of bone implants these are compounded by the necessity to ensure correct bone healing, a process heavily influenced by fluid dynamics and mechanical loading.^[^ ^228^ ^]^ Commonly used osteoblast 2D in vitro models can be applied to test an array of prospective molecules and materials, however, these fail to provide a model capable of mimicking the complex architectures subjected to dynamic chemical and mechanical changes vital to bone remodeling. Thus, in vivo models are generally accepted as a requirement for safety validation. The maxillary sinus floor augmentation and the capsule model are two examples of highly valued small animal models broadly employed in biomaterial evaluation.^[^ ^229^ ^]^ These aim to provide contact between wounded bone and a biomaterial while maintaining native tissue architecture in a living host, although fail to replicate mechanical stimulation, leading to a need for larger animal models dependant on the functionality of the implantable device. More importantly, in vivo model selection should be carefully considered with attention to the ratio of cancellous, cortical, or specialized bone, their microstructure, size, site, and vascularization. For instance, rodents and rabbits have limited similarities in macroscopic bone architecture, presenting higher bone healing capacity that can limit their extrapolation to the healing response of mature humans.^[^ ^226^ ^]^ Canines display a yearly trabecular bone turnover significantly higher than adult humans while mature sheep present denser and stronger trabecular bone subjected to seasonal bone loss.^[^ ^226^ ^]^ Coupled with variations between species, aging, genetic background, costs, and ethics limit their use as high throughput techniques.

#### 3.3.4 Future Perspectives for Osseointegration Analysis

A similar need can be observed in the pharmaceutical industry, which has pushed forward the development of novel models capable of replicating tissue architecture and cell‐to‐cell interactions to provide disease models.^[^ ^230^ ^]^ Some of the current techniques arising to overtake the high throughput 2D cell culture test common in pharmaceutics have been of interest for bone research with spheroids, scaffolds, cell culture sheets, bioreactors, or microfluidic devices applied to better understand bone healing.^[^ ^231^ ^]^ All of these aim to mimic specific bone microenvironments and, albeit promising, are still under development. In the case of self‐assembled cell aggregates or spheroids, their cost effectiveness and high throughput are superseded by the inhomogeneous nutrient and oxygen distribution alongside variations in size and shape.^[^ ^232^ ^]^ Scaffold architecture can cause limited nutrient uptake or cell adhesion at the core, leading to necrosis, or variations in cell behavior driven by substrate stiffness while microfluidics and organs‐on‐a‐chip require more testing to ensure reproducibility.^[^ ^232^ ^]^ As an alternative, other authors have showcased the importance of the immune system in the process of bone regeneration and the interactions between osteoblasts, osteoclasts, and macrophages not considered in conventional 2D cultures. Their roles in the immune response and bone formation have led to the development of assays in osteoimmunomodulatory (OIM) environments and co‐cultures trying to emulate the process of bone regeneration.^[^ ^233^ ^]^ Similarly to the benefits previously mentioned in the immune section, this is a change in paradigm that can potentially provide a representative model for material testing. These contrast with the pure chemical tests in the form of immersion in simulated bodily fluids (SBF) that have been available since the early 90s (ISO 23317). Over the years, it has become clear that the method developed by Kukobo^[^ ^234^ ^]^ is a fundamentally qualitative assay, requiring long times of incubation similar to cell cultures (1–4 weeks) to nucleate measurable deposits and fails to showcase the activity of moderate substrates.^[^ ^234^ ^]^ This has led to alternative in vitro tests arising over the last decade based in the enzymatic deposition of calcium phosphate or simple titration to analyze the bioactivity of materials. From these, the recent method of Zhao et al.^[^ ^235^ ^]^ has demonstrated a good correlation with common in vivo models, potentially providing a simple and quick, ≈4–5 h, platform that can be used to rapidly evaluate biomaterials. With the end purpose of providing a rapid high throughput method to evaluate novel alloys, this technique alongside cell cultures in OIM environments seems to be a potential candidate that, coupled with more mature spheroids, scaffolds, microfluidics, and other platforms, could revolutionize the testing of novel alloys.

### 3.4 Bacterial Colonization, Biofilm Formation, and Antimicrobial Metals

Statistics show that infection risks in the prosthetic field are relatively rare as a result of modern surgical techniques and antibiotic prophylaxis, accounting for just 1–2% of all hip and knee joint replacements.^[^ ^236^, ^237^ ^]^ Nevertheless, hospital‐acquired infections in the US alone accounted for 2 million cases of which 50–70% were associated with medical devices.^[^ ^238^, ^239^, ^240^ ^]^ In the case of an infected device, the usual gold‐standard course of action involves a two‐stage revision where the compromised implant is removed alongside debridement of the surrounding tissue and short‐/long‐term antibiotic treatment followed by a second surgery with a new device once the infection has been cleared.^[^ ^241^ ^]^ Such courses of action result in a reduction in the patient's prospective recovery with infection rates reaching between 30 to 50% in subsequent surgeries.^[^ ^242^ ^]^ The increased surgery time, costs to healthcare service, and reduced quality of life for the patient renders infection of an implantable device a bleak scenario for patients, healthcare providers, and implant manufacturers. It does, however, present a significant impact opportunity for a novel antimicrobial alloy system.

#### 3.4.1 Fundamentals of Bacterial Colonization and Biofilm Formation on Implants

Once a medical device has been implanted, an active competition between native tissue and pathogens to integrate on the surface will take place. If host cells establish on the surface first, any potential microorganism will find a cell layer with a functional immune system that can counteract bacterial adhesion and proliferation.^[^ ^243^, ^244^ ^]^ In contrast, once bacteria attach to a surface they develop a highly complex community, resulting in a 3D aggregation of cells surrounded by a protective layer of extracellular polymeric substances (EPS). This biofilm raises the ability of microorganisms to tolerate antimicrobial substances a hundred fold, complicating their treatment and resulting in a positive vector for antimicrobial resistance (AMR) development.^[^ ^37^ ^]^ An ideal response should therefore contemplate the early stages of bacterial mass transfer, attachment, proliferation, and biofilm formation, with a prospective novel alloy being capable of modulating and preventing bacterial interactions.

**Figure 3:**
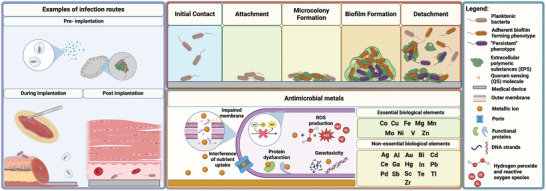
Examples of infection routes alongside stages in biofilm formation and antimicrobial mechanisms antimicrobial elements.

Several studies have shown that surgical site infections can be ascribed to patient‐specific or endogenous (e.g., coexisting infection, age, or diabetes) and external or procedural (e.g., inadequate sterilization of medical equipment, poor skin disinfection or antibiotic prophylaxis) factors giving a plethora of routes for bacterial attachment (**Figure** **3**).^[^ ^245^ ^]^ Bacteria can be transported to a surface by aerosols or sedimentation/diffusion if in an aqueous solution and through both active movement and/or long‐range forces. Chemotaxis^[^ ^246^, ^247^ ^]^ and long‐range, >50 nm, nonspecific forces (gravitational, van der Waals, electrostatic and hydrophobic interactions) guide these microorganisms to a potential binding surface,^[^ ^248^, ^249^, ^250^ ^]^ which are weakly and reversibly attached through, specific short‐range, <5 nm, forces.

After the microorganisms have adhered to the surface, the bacterial cells will proliferate in an accumulation phase, firstly by the creation of microcolonies that will expand to form macrocolonies enclosed in a rich EPS matrix (Figure 3). This layer is composed of polysaccharides, nucleic acids, proteins, and lipids, and acts as a protective barrier while facilitating nutrient capture and dissemination.^[^ ^251^, ^252^, ^253^ ^]^ More relevant is the coordinated effort to regulate self‐organization and the overall behavior of these cell aggregations. This mechanism is referred to as quorum sensing (QS) and is based on self‐induction where an increase in population will lead to a raise in bacteria‐dependent molecules that, in turn, will promote changes in gene expression.^[^ ^241^, ^254^ ^]^ By the effective use of QS these communities can regulate sporulation, biofilm production, conjugation, motility, and virulence factors, resulting in a competitive advantage over planktonic cells.^[^ ^247^ ^]^ These benefits continue to promote bacterial growth and biofilm maturation, however, as the population becomes larger, available nutrients and oxygen become scarce while metabolic by‐products and toxic waste increase. This results in the triggering of a new QS response and secretion of molecules that reduce the structural integrity of the EPS matrix, followed by cell detachment both in the form of planktonic cells and micro‐/macrocolonies.^[^ ^241^, ^255^ ^]^ Thus, the matured biofilm becomes a new vector for infection of the medical device and host tissue. This is compounded by microbial cells retaining their highly resistant and adherent biofilm phenotype or, in the case of starved bacteria, “persistent cells” that can lay dormant during prophylactic treatment, resurfacing as new pathogens once antibiotic therapy halts.^[^ ^256^, ^257^ ^]^

#### 3.4.2 Potential Antimicrobial Metals

Based on the catastrophic consequences that biofilm formation can have on the medical device industry and the patient, it is paramount to develop and implement techniques that address its prevention from bacterial adhesion to biofilm maturation and cell detachment. Metallic ions possess numerous antimicrobial mechanisms specific to each element, making them effective alternatives to treat infection (Figure 3). Active transition metals such as As, Fe, Cu, Cr, Co, V, Te, or Ni can catalyze electron transfer with metabolic by‐products (e.g., H_2_O_2_ and O_2_^−^) to increase the presence of Reactive Oxygen Species (ROS) through Fenton chemistry.^[^ ^258^, ^259^ ^]^ Other groups have showcased the ability of metallic ions to disrupt or destroy [4Fe‐4S] protein ligands with subsequent active iron release and ROS formation.^[^ ^260^, ^261^ ^]^ Moreover, ROS can arise from the thiol‐mediated reduction of metallic ions stemming from their affinity with sulfur which, in turn, can result in thiol reserve depletion.^[^ ^262^ ^]^ In contrast to ROS production, protein dysfunction can lead to cell toxicity with metallic elements reported to interact with Fe‐binding sites, destroy Fe–S clusters, or influence other catalytic and structural metals, e.g. Pb(II), Ni(II), or Ag(I).^[^ ^263^ ^]^ Other ions have showcased an ability to interfere with cell membrane structure and functions with Ag compromising the outer membrane and disrupting the electron transport chain,^[^ ^264^, ^265^ ^]^ or Cu and Cd affecting the integrity of cell envelopes through lipid peroxidation.^[^ ^266^ ^]^ Cr and Ga have been linked with cell starvation due to their ability to prevent S and Fe uptake, respectively, while Mn, Cr, Co, Cd, Mo, Sb, and As have been shown to promote DNA damage.^[^ ^263^ ^]^ All these mechanisms result in a highly effective mode of action that has led to a rise in interest in harnessing metallic elements to tackle AMR through surface treatments of the base device.^[^ ^267^, ^268^, ^269^, ^270^, ^271^ ^]^ Besides the already mentioned elements, the need for a greater diversity of antimicrobials and the increased risk of AMR is rapidly expanding metals explored as antimicrobial candidates, including Al, Au, Bi, Ce, Ga, Mo, Pd, Pb, Sn, Te, Tl, Zn, or Zr.^[^ ^272^, ^273^, ^274^, ^275^ ^]^ Elements with established use and comparatively low toxicity, such as Ag and Cu, have become prevalent in the biomedical field, whilst those with well‐established bioaccumulative toxicity such as mercury, arsenic, and antimony, historically used in agriculture and medicine, have become less prevalent.^[^ ^263^ ^]^

When metallic elements are considered from a biological perspective, they can be either fundamental to various biochemical processes or non‐essential metals. Electron transfer, catalysis, cell membrane, and DNA structures are only a few of the organic processes where metals are indispensable and potentially lethal to all cells in increased doses.^[^ ^263^, ^276^ ^]^ On the other hand, silver (Ag), mercury (Hg), or tellurium (Te) are a few non‐indispensable metals that are extremely poisonous to a broad spectrum of organisms at relatively low concentrations.^[^ ^263^, ^277^ ^]^ The ability of these elements to interfere with biological processes stems from three main properties: donor atom selectivity, reduction potential, and speciation. The ability of metal ligands to selectively interact with specific biomolecules is conferred by a preference for donor ligands following the Irving–Williams series (Mn(II) < Fe(II) < Co(II) < Ni(II) < Cu(II) > Zn(II)).^[^ ^272^, ^276^, ^278^ ^]^ However, when homeostasis is altered, metals at the high end of the scale can bind to ligands requiring less competitive metals, altering protein structure and function.^[^ ^263^ ^]^ Similarly, soft acids (e.g., Hg(II), Cu(I), Ag(I) and Cd(II)) and borderline acids (Co(II), Ni(II), Cu(II) and Zn(II)) tend to strongly interact with soft bases in the form of protein sulphhydryl (R‐SH) groups, with their toxicity towards planktonic cells relating to their affinity with sulfur.^[^ ^279^, ^280^ ^]^ On the other hand, a lack of selectivity can lead to “ion mimicry” where cellular dysfunction is developed over the incorrect binding of an ion metal with a similar structure to the required protein cofactor.^[^ ^263^ ^]^ Besides these interactions, metallic elements are characterized by their ability to acquire electrons. This tendency, thermodynamically defined by their reduction potential, reflects the reactivity of such species with biological molecules and roughly correlates with antimicrobial efficacy.^[^ ^277^, ^279^ ^]^ Altogether, these three parameters govern reactivity of metallic species which can be potentially used to guide the development of novel alloys for antimicrobial applications.

#### 3.4.3 Antimicrobial Tests for Biomaterial Assessment

Ideally, tests developed to understand the antimicrobial effect of novel alloys on multicellular organisms should try to mimic the natural infection and biofilm formation processes, making the subcutaneous foreign body, intraperitoneal foreign body or the osteomyelitis in vivo infection model highly relevant.^[^ ^281^ ^]^ The low availability, high costs, required expertise, and ethical implications of in vivo models render these techniques more useful in the later stages of biomaterial development and safety assurance. In contrast, in vitro studies in the form of agar diffusion, macro/microtiter, or direct inoculation as stated in ISO 22196 tend to be the preferred assays used for the early screening of antimicrobial materials.^[^ ^282^, ^283^ ^]^ These assays quantitatively measure the antimicrobial activity of a prospective material by ensuring direct or indirect contact with a culture of the desired bacterium. Direct contact macro/microtiter methods, modified for solid surfaces, submerge the desired sample in a bacterial culture while ISO 22196 disperses droplets of these cultures ensuring full contact through the use of aseptic covers.^[^ ^283^ ^]^ Indirect techniques such as the agar diffusion test or variations of well diffusion assays quantify the inhibition halo, created by antimicrobial elements leaching from a sample into a semi‐solid nutrient platform where a bacterial culture has been previously inoculated.^[^ ^284^ ^]^ Their simplicity makes these techniques of interest for the rapid evaluation of numerous materials and their interactions with other antibiotics, however, some of these assays require labor‐intensive processes and only provide an endpoint perspective of the material/bacterial interactions. With dozens of potential antimicrobial candidates for a multi‐composition alloy affected by element percentage, microstructure, and bacterial species,^[^ ^273^, ^283^, ^285^ ^]^ their application as high throughput methods seems challenging. In this regard, the biomaterials community should implement more novel approaches with the aid of variations on microtiter assays that with the use of turbidity or fluorescence markers can provide growth curves for hundreds of samples in the same experiment. When used as input for simple mathematical models (e.g., Gompertz model) these could rapidly select potential candidates for further study, an approach already proposed by Brochado et al.^[^ ^286^ ^]^ to analyze antibiotic interactions to offer a platform to guide the clinical use of antibiotic therapies. Longer tests to account for biofilm formation should also become more broadly applied with standardized and commercialized approaches in static, e.g., the Calgary device, and dynamic, e.g., the Robbins biofilm sampler or the drip flow reactor (DFR), models already available.^[^ ^287^ ^]^ As the technology evolves, genomic systems may become of interest for their application,^[^ ^287^ ^]^ but with the numerous variables affecting a prospective antimicrobial alloy, the community should first strive for the development and standardization of high throughput methods.

#### 3.4.4 Future Considerations for Antimicrobial Alloy Development

With the main objective to provide bioactive materials, novel alloys should be tested both in the short and long term, as complex interactions may not be apparent initially. For instance, W and Co mixtures have been shown to enhance ROS leading to higher toxicity levels^[^ ^288^ ^]^ while the selectivity of other antimicrobial metals, like Se,^[^ ^289^, ^290^ ^]^ may require combinations of other elements to provide high spectrum coverage. On the other hand, it is arguable that in the foreseeable future antimicrobial elements will be used alongside common antibiotic therapies that can disrupt AMR development. Some short‐term studies have revealed the ability of some of these combinations to result in synergistic effects,^[^ ^291^, ^292^ ^]^ however, long‐term studies also indicate that these benefits could be overshadowed by enhanced AMR development in the long term.^[^ ^293^ ^]^ Similarly, genomic mutations resulting in de novo generation of resistance mechanisms to antimicrobial metals are rare, but genes encoding the capture and efflux of heavy metals have been developed due to environmental exposure, which can be transferred to clinical settings.^[^ ^272^, ^294^ ^]^ More concerning is the correlation that has been found between heavy metal exposure and antibiotic resistance development with subtoxic levels of Zn or Cu being only two examples of heavy metals involved in co‐selection and proliferation of AMR.^[^ ^295^ ^]^ Thus, design criteria should be expanded to encompass long term analysis and combinations of metals and antibiotic molecules, pushing forward new high throughput methods to test effectiveness and impact in AMR.

### 3.5 Summary

Understanding the intricate biological reactions triggered by implanted devices is paramount. By identifying the key proteins and molecules that facilitate these reactions, we are able to promote the design of alloys that foster optimal wound healing, angiogenesis, osseointegration, and antimicrobial response. **Table** **1** outlines some of these essential proteins and molecules, along with examples of conventional in vitro and in vivo methods for assessing the biological responses to these alloys.

**Table 1:** Summary of proteins and molecules that have beneficial effects on key biological systems that respond to implanted devices along with methods of evaluation.

Biological process	Beneficial protein and molecules	Conventional methods for evaluation	
Inflammatory Response	Fibrin	In Vitro	Neutrophil based assays
	Fibronectin		Lymphocyte proliferation test
	Basic fibroblast growth factor (bFGF)		Macrophage polarization
	Vascular endothelial growth factor (VEGF)		
	M2 macrophage markers (e.g., TGF‐β, CCL24, IL‐10, CD206)	In Vivo	Patch test
			Implantation assays (e.g., Subcutaneous, intraperitoneal)
Angiogenesis	Fibroblast growth factors (FGFs)	In Vitro	Direct cell counting
			Metabolic assays
	Platelet derived growth factors (PDGFs)		Cell cycle staining
			Scratch/Boyden chamber assays
	Thrombin		Tubulogenesis assays (e.g., Matrigel)
		In Vivo	Chick chorioallantoic membrane
	Hypoxia‐Inducible Factor (HIF‐1)		Dorsal air socket model
			Subcutaneous implantation
	Vascular endothelial growth factor (VEGF)		Plug assays
			Chamber test
			Zebrafish
		*Ex vivo*	Tubulogenesis of aorta explants
Osseointegration	NF‐kB (RANK/RANKL)	In Vitro	Osteoblast 2D models (e.g., proliferation, differentiation, mineralization)
	Osteoprotegerin		
	Osteocalcin/Osteopontin/Runx2		Simulated bodily fluid assays (e.g., Kukobo's, enzymatic, titration)
	Type I collagen		
	Bone sialic acid glycoprotein	In Vivo	Maxillary sinus floor augmentation
			Capsule model
	Alkaline Phosphatase (ALP)		Other bone defect models (e.g., tibial, orbital, etc)
Bacterial colonization and biofilm formation	Normally defined by a reduction in bacterial population	In Vitro	Agar diffusion
			Macro/microtiter
			Direct inoculation
			Calgary device
			Robbins biofilm sampler
			Drip Flow reactor
		In Vivo	Subcutaneous foreign body
			Intraperitoneal foreign body
			Osteomyelitis model

## 4 Methods to Accelerate Biomedical Alloy Development: Matching the Pace of Discovery from Modeling to Manufacture and Validation

When considering the design space of potential novel bioactive titanium alloys, there exists a plethora of metalloids, nonmetals, transition metals, rare/alkali earths, and other metals of interest. There are between 30–40 candidate elements within the periodic table that may be of interest to incorporate for tuning microstructure and biological functionality resulting in ternary, Ti‐Nb‐Fe, to quinary, Ti‐Nb‐Mo‐Zr‐Sn, or even more complex multi‐element systems, Ti‐Nb‐Ta‐Zr‐Fe‐O‐Si.^[^ ^54^, ^296^, ^297^ ^]^ This leads to an upper bound of ≈7.6 × 10^5^ prospective multicomponent systems that require compositional optimization and lengthy microstructural tunning to achieve target requirements. With potentially tens to hundreds of compositional and thermomechanical combinations per system, exploring such a large design space in a reasonable timeframe while reducing R&D costs becomes a daunting process.

To effectively explore this vast design space and optimize for an expanded list of alloy criteria it is critical to align the pace of discovery, manufacturing, and validation. Generally, material discovery has greatly benefited from Integrated Computation Materials Engineering (ICME) and alloy design approaches that use thermodynamic databases to narrow large compositional space and investigate the effect of alloying elements on microstructure and properties. Such methods have already been introduced into other areas of alloy discovery, such as the aerospace industry. However, these perform poorly when extrapolating to new materials systems that are not covered by reliable thermodynamic databases.^[^ ^298^ ^]^ In addition, databases for biological responses to metallurgical phases simply do not exist with data collated from different studies difficult to compare given the variety in methods and measurements across studies.^[^ ^298^, ^299^, ^300^, ^301^ ^]^ By using modeling techniques to enable the rapid generation and discovery of appropriate alloys, we can down‐select the quantity pushed forward for physical experimentation. Maintaining this pace beyond mapping new alloy design spaces is essential for translation. As such, this section reviews both feasible manufacturing routes but also high throughput biological validation approaches that will be critical to transforming the biomedical alloy mindset.

### 4.1 Physical Testing vs Modeling Approaches to Facilitate Alloy Discovery

In the United States, the Materials Genome Initiative (MGI) was announced in 2011 as a federal multi‐agency initiative to increase the rate of materials discovery and design. Through the approach, new datasets were generated and shared comprising vast numbers of materials, enabling researchers to collaborate more successfully and efficiently.^[^ ^302^ ^]^ High‐throughput modeling methods have commonly been used in the discovery of high entropy alloys (HEAs), normally applied to the aerospace sector due to their ability to tolerate high operating temperatures.^[^ ^303^, ^304^ ^]^ High‐throughput modeling approaches have proven effective at obtaining desired microstructures and properties, improving the rate at which candidate aerospace materials have been identified.^[^ ^304^ ^]^ These approaches have led to many key advancements within materials science, across multiple research areas, from shape memory alloys, to organic light‐emitting diodes (OLED) and polar metals. The wider integration of efforts and improved databases with enhanced structure‐function relationship knowledge will enable a greater understanding of the materials genome, thus leading to more advanced developments.^[^ ^302^ ^]^

Furthermore, since the MGI, there has been a much greater emphasis on the necessity of rapid generation and use of open‐source databases to accelerate the development of metal alloys, in particular HEAs.^[^ ^304^, ^305^ ^]^ Such databases can take multiple forms, and contain either simulated data about structures and compounds, or experimental property data for bulk materials. These databases can then be interrogated by machine learning or even simpler computer‐aided methods to discover materials and predict their properties.^[^ ^304^, ^306^ ^]^ In addition, computational methods, such as first principles simulations, molecular dynamics, and CALPHAD (CALculation of PHAse Diagrams), are becoming increasingly more common in alloy discovery efforts. One major benefit of using computational predictions is the large reduction in cost compared to experimental approaches.^[^ ^304^ ^]^

From a biological perspective, machine learning opens a door to unearth complex relationships between alloying elements and biological functionality that could be exploited to develop materials capable of guiding the wound healing process and provide new functionality, for example, antimicrobial effectiveness. Nevertheless, the number of potential targets, lack of unified databases, and our limited understanding of the effect novel elements have on these complex processes pose a barrier to their implementation. In the previous section, it has become clear that alloy development should offer a set of properties that are similar to the operation temperature and specific strength in aerospace can be used to incrementally improve biomedical titanium alloys. Instead of a binary space centered on biocompatibility and mechanical performance, immunomodulation, vascularization, osseointegration, and antimicrobial properties should be included as critical objectives in a relevant model. Nevertheless, the complexity of these processes and conventional simplified tests reveal that several methods may be required to assess and predict biological outcomes. On the other hand, some of the new changes in paradigm that could offer a more relevant view of specific properties have only been recently developed. For instance, macrophage polarization or the titration method developed by Zhao et al.^[^ ^235^ ^]^ can be powerful tools to assess materials, albeit would require further investigation to ensure their usefulness as biological predictive markers. Then, it must be considered that the available data is restricted to already established materials, which given the exploratory nature of alloy discovery implies a critical need for big training datasets. This should be coupled with the broad spread of these novel techniques or, even if using mature methods, the need to standardize experimental setups to enable collaboration. Adding our growing knowledge of the role of metals in biological processes, it is clear that building a predictive platform for alloy discovery will pose a global challenge. Still, the prospective benefits to healthcare and society should ensure that the biomaterials community takes a step forward to tackle this obstacle. For this reason, this manuscript offers a series of properties that should be observed as objectives to be included for the development of active metal devices, while including recommendations on available techniques both in manufacturing and testing to feed a prospective model.

### 4.2 Manufacturing Approaches to Support High‐Throughput Alloy Discovery

**Figure 4:**
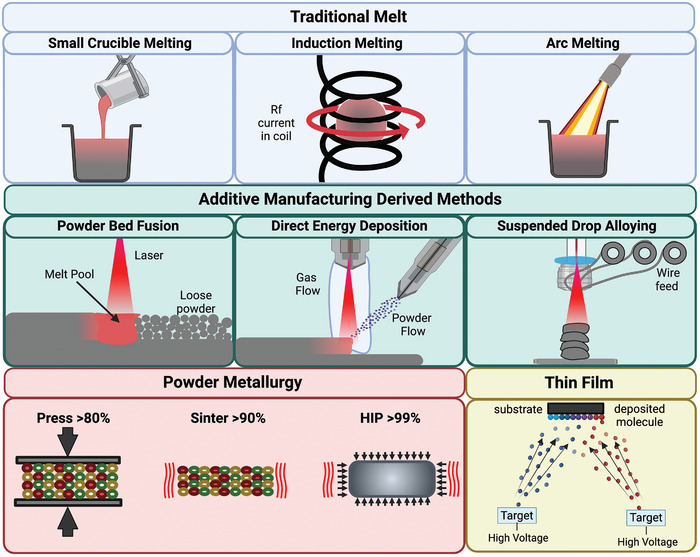
Schematic of traditional melt, additive manufacturing, powder metallurgy methods, and thin film methods to facilitate alloy discovery.

High‐throughput combinatorial techniques that can rapidly synthesize different alloy compositions, structures, and phase distributions, are essential. Such techniques must be able to produce specimens adequate for biological testing at a rate high enough to allow for several element combinations to be tested in a short timeframe. Traditional melt and powder metallurgy methods will be discussed alongside thin‐film deposition and in situ alloying by additive manufacturing (AM) (**Figure** **4**). Each method has its own benefits and limitations highlighting the need for a variety of practical tools. Such combinatorial techniques have been previously applied to other industries, for example, the pharmaceutical industry often employs modeling software to produce compound libraries, to improve the research's discovery phase. Similar methods are becoming increasingly utilized in the discovery of novel inorganic materials. However, the aim is to combine the rapid approaches seen in the pharmaceutical industry with the screening techniques used in materials discovery.^[^ ^307^ ^]^ Increasing the rate of discovery whilst reducing costs is a key focus within materials development. In particular, alloys tend to have diverse and often complicated composition‐microstructure interlinks, and so the manufacture and characterization of novel alloys is often an expensive and lengthy process.^[^ ^304^ ^]^ This is especially true when, in addition to considering key physical properties, one must also consider the critical biological responses reviewed in the previous section.

#### 4.2.1 Traditional Alloy Development Approaches

The main objective of composition design is to select and control the microstructure and phases manifested by a metal component at the microscale knowing the process by which it will be manufactured. This includes control of grains’ size and morphology, as well as the distribution of these phases.^[^ ^308^ ^]^ Aside from the field of HEAs, conventional alloys are typically underpinned by a binary or ternary system with minor additions of elements to refine the microstructure or enhance certain properties. Nickel superalloys used for aerospace applications are largely based on binary systems (namely Ni‐Al), despite having several other elements listed in their compositions. These minor alloying elements allow precise control of the microstructure, conferring additional corrosion resistance and strength through mechanisms such as solid solution strengthening and precipitation hardening.^[^ ^57^ ^]^ The addition of minor alloying elements to improve material properties has been a successful strategy in the development and refinement of metallic materials such as steels, Mg‐based alloys, Al‐based alloys, and binary intermetallic compounds.^[^ ^309^ ^]^ However, this strategy has not been as widely adopted in developing new biomedical alloys.

Among the most highly engineered alloys, most tend to include 5–10 alloying elements, which makes it challenging to isolate the contribution of individual or combinations of elements to microstructural and phase stability. Traditionally alloy development relied on an iterative “one discrete composition at a time” approach. After an initial composition is selected, manufactured, and analyzed, the composition is tweaked as deemed appropriate and the process is repeated. This iterative strategy is costly and time‐consuming, even when using research‐scale, small‐volume manufacturing methods. As the properties of an alloy are determined by its chemistry and microstructure, comprehensive characterization of new compositions should include numerous samples processed in a range of conditions (e.g., processing parameters, heat treatments cycle, surface treatments) and geometries (e.g., tensile, fatigue, creep). The numbers of samples required for characterization also tend to rapidly increase with the complexity of the alloy and the number of alloying elements it includes. Considering basic characterization of a binary composition following this traditional approach can take months, developing a novel alloy with more than 5 alloying elements can take several years.

Some small‐scale ingot production technologies exist that lend themselves favorably to a research‐focus framework, such as vacuum arc melting and induction melting, which can produce small metal ingots in the range of 3–500 g, at a rate of 1–10 ingots per day. These “melt” methods are reliable but can be slow, with ingots often needing several remelts to achieve compositional homogeneity.^[^ ^310^, ^311^ ^]^ Methods using a crucible also open the risk of contamination and interactions with crucible materials.

Alternatively, powder metallurgy (PM) processing is recognized as a route for small pellet alloy production through either cold press + sinter, or cold press + sinter + hot isostatic press (CHIP) methods.^[^ ^312^ ^]^ With the right workflow, these may provide a higher throughput than the melt methods as the small particle size and correctly selected sinter conditions limit the possibility of large segregation whilst enabling alloying through solid‐state diffusion. Likewise, the lack of a liquid stage eliminates crucible interactions.

#### 4.2.2 Thin Film Combinatorial Metallurgy

Thin film sample fabrication is well established using several methods: physical vapor deposition,^[^ ^313^ ^]^ chemical vapor deposition,^[^ ^314^ ^]^ electrochemical methods,^[^ ^315^, ^316^ ^]^ and ion implantation.^[^ ^317^ ^]^ Deposition is typically on a silicon wafer (≈100 mm dia.) up to a thickness of several hundred nm. Advances in deposition techniques have improved the control of the process, improving the accuracy of deposited layers and the corresponding compositional control.^[^ ^318^, ^319^ ^]^ Films may be deposited using either continuous or multilayer methods. During continuous co‐deposition, simultaneous sputtering of the material allows for atomic mixing, however, the available compositional gradients are limited by the sputtering rates of each element. Multilayer deposition is slower, with each element being deposited in nanoscale layers, but allows for much greater compositional control. For alloy development, it is likely that deposits using either method would require further heat treatment to produce a more representative crystalline structure.^[^ ^320^ ^]^ However, the primary appeal of these methods lies in their capacity to generate thousands of compositions in a single day, making them an excellent tool for property screening.

The use of thin‐film combinatorial methods for alloy discovery has often focused on applications requiring a specific physical response that can be easily measured and mapped across the design space. These include shape memory alloys, transducer materials, magnetostrictive alloys, and materials used in energy storage applications.^[^ ^321^ ^]^ From a medical perspective, thin film techniques are primarily employed to create surface coatings aimed at enhancing biocompatibility, corrosion resistance, mechanical properties, or other criteria driven by end‐user requirements.^[^ ^322^ ^]^ Some studies have also mapped composition and antimicrobial efficacy across compositionally graded thin‐film specimens showing potential for this as a method of alloy discovery for specific biological functionality.^[^ ^323^, ^324^ ^]^

The gradient length scales and microstructural differences between film and bulk alloys suggest that it should be used cautiously to inform further developments rather than as a single‐step solution. However, with its ability to produce compositional grading and subsequent mapping of biological response across this surface, thin‐film deposition is a promising route for guiding functionally driven biomedical alloy discovery.

#### 4.2.3 Additive Manufacturing Derived Methods

AM systems can be classified by how feedstock is supplied: powder bed systems, powder feed systems, and wire feed systems.^[^ ^325^ ^]^ Each of these relies on an energy source, often a laser, locally melting feedstock material in either powder or wire form. Given the local melting, mixing, and solidification during the fabrication process, these technologies not only enable netshape fabrication, but also the potential for in situ alloying by using multiple feedstock materials at once. Composition changes are achieved relatively simply by varying material ratios; in the case of wires by adjusting the feed rate, and for powders by adjusting the blended ratio of elements.

##### Laser Powder‐Bed Fusion (PBF‐LB)

Powder bed fusion (PBF) is the most utilized AM technology for the manufacturing of metal components. Both laser and electron beam can be used as the melting energy source, but for alloy development, laser is typically preferable. The high‐powered laser is used to melt and fuse the metal powders, in a layer‐by‐layer approach, according to CAD programming.^[^ ^326^ ^]^ Electron beam processes require a vacuum atmosphere which can result in preferential volatilization of higher vapor pressure elements during melting and alteration of the final material composition,^[^ ^327^ ^]^ whereas the inert gas atmosphere used in PBF‐LB helps suppress evaporation.

Whereas traditional AM via PBF‐LB uses pre‐alloyed power feedstock, in‐situ alloying typically uses mixed/blended, or mechanically alloyed powder. This allows for relatively low‐effort investigation of a new alloy system by preparing different blends, using powders of the constituent elemental powders rather than dedicated atomization of pre‐alloyed material, which is a costly endeavor. Simple mixing is a low‐cost and pragmatic process for blended powders, however, it has been associated with local chemical inhomogeneity where the degree of mixing is not comparable to the melt‐pool size (≈100 µm typically).^[^ ^328^ ^]^ Likewise, it is generally advised that powders have a similar particle size distribution as broad distributions that include fine particles are known to flow and spread poorly during PBF‐LB.^[^ ^329^ ^]^ A recent variation, satellite mixing, however, involves intentionally decorating the surface of a base spherical elemental powder with very fine alloying elements. This has been shown to improve chemical homogeneity in the alloyed material, although the flowability of the satellite mixed powder was poorer than the carrier powder.^[^ ^328^ ^]^ Mechanical milling without alloying presents another way to reduce chemical inhomogeneity by increasing powder contact area although changes to the powder morphology, away from the ideal “spherical”, again present potential flowability difficulties.^[^ ^329^ ^]^

During processing, key variables such as power, speed, hatch spacing, and layer thickness govern many aspects of the as‐built material microstructure and properties.^[^ ^305^ ^]^ Inhomogeneity and segregation may be reduced by increasing energy input and thus the melt pool temperature, size, and Marangoni forces which drive melt‐pool mixing.^[^ ^330^ ^]^ Likewise, materials with different melting points, heat capacity, enthalpy of fusion, and reflectivity require that energy input is high enough to melt the least favorable of the blended elements to avoid unmelted particles trapped in the microstructure. This energy increase can lead to the “unmelted particle‐keyhole” dilemma whereby energies required for complete melting can lead to keyhole pore formation.^[^ ^329^, ^331^, ^332^ ^]^ It is also likely that parametric studies will be necessary for significant changes in the laser/powder interactions that accompany compositional blend changes. Depending on the system, there may be no appropriate parameter window giving a fully dense material without segregation. Laser rescanning each layer has proven effective at reducing segregation in certain cases.^[^ ^333^ ^]^ Using a post‐processing heat treatment to homogenize material through diffusion has also been demonstrated as effective.^[^ ^334^, ^335^ ^]^

Despite these technical challenges, the use of in situ alloying via PBF‐LB has been widely demonstrated for Ti alloys.^[^ ^335^, ^336^, ^337^, ^338^, ^339^, ^340^ ^]^ The simplicity of feedstock preparation and the flexibility of final specimen geometry make it an attractive tool for investigating alloy systems.

##### Laser Beam Direct Energy Deposition (DED‐LB)

Whilst both LPBF‐LB and DED‐LB use a powder feedstock and laser energy source, DED‐LB delivers both via a CNC (or robot) mounted processing head. A high‐powered IR‐laser generates a melt pool on the substrate or previously deposited layers and inert gas streams “blow” powder into the pool to deposit material. Powder is typically fed either co‐axially or from a discrete aperture(s) off‐axis to the beam. Structures are built up layer‐by‐layer with the melted bead an order of magnitude larger than PBF‐LB (≈0.5–3 mm). The use of DED‐LB for titanium‐based in situ alloying Is widely reported.^[^ ^341^, ^342^, ^343^, ^344^, ^345^ ^]^

Whilst it is possible to use blended powder feedstock in the DED‐LB process, a distinct advantage of DED‐LB over PBF‐LB is that different hoppers can feed simultaneously depositing nozzles, removing the need to pre‐blend.^[^ ^343^, ^346^ ^]^ Composition is adjusted by varying the individual powder feed rates. Several studies have used this to generate compositionally graded structures.^[^ ^347^ ^]^ For medical alloy development, a single specimen with a compositional gradient could be used to rapidly assess how biological response changes with composition in an efficient manner.

Though a larger melt‐pool and no need for pre‐blending avoids some of the drawbacks of in‐situ PBF‐LB alloying, DED does present some technical challenges for alloy development. Powder capture rate, the percentage powder captured by the melt pool compared to the powder delivered to the nozzle, is always <100% and varies significantly with material, system, and nozzle arrangement.^[^ ^348^ ^]^ Different materials are likely to be subject to different capture rates depending on their physical properties. Additionally, incoming powder velocity to the melt pool has been shown to be a critical in determining capture rates. At high velocities, the residence time of the particle on the melt pool surface is short and a “rebounding” effect has been observed under high‐speed photography.^[^ ^349^ ^]^ Li et al.^[^ ^350^ ^]^ reported that ideally:

(1) $$\frac{d_{1}}{d_{2}}=\sqrt{\frac{\rho_{2}}{\rho_{1}}}$$

where d and ρ are the particle diameter and density for the two powder materials being delivered. By maintaining this ratio, the powders should travel at the same velocity in a combined gas stream. This should reduce the chance of inflight segregation of particles. These challenges in achieving a known and predictable powder capture are magnified further is mass flow rates are varied along the build height to create a known compositional gradient. Likewise, the specific nozzle arrangement may inadvertently create a composition gradient across the melt pool that is unintended if different elements are supplied along different flow vectors.^[^ ^348^ ^]^ Despite the practical challenges, the benefits of compositional grading for rapid determination of biological response thresholds make DED‐LB an attractive option for rapid medical alloy exploration.

##### Suspended Droplet Alloying (SDA)

While not widely adopted, the suspended droplet alloying method provides a noteworthy alternative to powder feedstock methods.^[^ ^351^, ^352^ ^]^ Derived from wire‐fed DED AM techniques, elemental wires are directly fed into a laser beam, producing a metal droplet that remains suspended via surface tension on the feeding wire, as shown in Figure 4. When the droplet size has grown sufficiently it drops, slowly forming a small sample. The size and lengthy molten duration compared to other AM methods provides good mixing. Composition is easily controlled using wire feed rates with 100% capture efficiency making the resulting material composition well controlled. There is potential for functional grading, however, given that specimens form drop by drop, any compositional gradient would likely form discrete layers, rather than continuous.^[^ ^351^ ^]^

#### 4.2.4 Summary of Methods

With the number of elemental combinations and an equally broad potential for phase precipitation, size, and morphology within each one, rapid alloy development by experimental means is often a difficult process. The methods outlined here aim to speed up this discovery phase by providing specimens that can be produced more rapidly than traditional methods and act as a base for biological evaluation. Each process has characteristic features, pros, and cons that should be appreciated; these are summarized in **Table** **2** below:

**Table 2:** Summary of the advantages and disadvantages of different combinatorial techniques.

Process	Pros	Cons
Melt methods (e.g., vacuum arc, induction etc.)	Good bulk compositional control Homogenous composition may be achieved	Time‐consuming and labor‐intensive Multiple melts may be required for homogeneous composition No compositional grading
PM methods	Relatively simple compositional control Solid state process Bulk homogeneity	May require HT for diffusion at the µm length scale Time‐consuming sintering HT steps Limited specimen size No Compositional grading
Thin film methods	Compositional gradient and control within single specimen Homogeneous material	Specimen size/thickness limited Requires HT for representative crystalline structure.
PBF‐LB	Ease of compositional control Specimen size/shape flexibility Good bulk homogeneity	May require HT for diffusion at the µm length scale Difficulties processing dissimilar materials May require process optimization. No compositional grading
DED‐LB	Ease of compositional control and grading Specimen size/shape flexibility	Powder capture limits compositional accuracy Potential for segregation when co‐feeding dissimilar powders May require homogenization HT
SDA	Ease of compositional control 100% material capture gives accurate composition control	Limited specimen size/shape Requires highly specialized equipment May require process optimization Compositional grading in discrete steps

### 4.3 High‐Throughput Biological Validation Approaches

This section has highlighted innovative modeling approaches that allow for rapid design space scanning and relatively rapid manufacturing methods that enable the physical production of alloy compositions and microstructures of interest. Beyond this, it is critical to align this capability with high‐throughput approaches that enable validation of target alloy characteristics, from mechanical to biological. Specifically focusing on assessing the biological impact of a large number of candidates, the pharmaceutical industry faces similar challenges with drug discovery with potential limitless design spaces of moieties and molecule combinations for disease treatment.^[^ ^353^, ^354^ ^]^ This sector has been transformed partly by high throughput screening (HTS) methods, which make it possible to test 20 000 compounds per week and even up to 100 000 compounds per day with ultra‐high throughput screening (UHTS).^[^ ^355^ ^]^ Herein we reflect on the lessons learned in drug discovery and in the development of other biomaterials classes to reveal critical steps required for rapid alloy validation.

HTS in the drug discovery process is heavily reliant on combinatory chemistry and robotics, which in turn are dependent on miniaturization. Generally, 2D cultures in 384 and 1536 well plates are the preferred systems for rapid screening of materials^[^ ^356^ ^]^ with some more novel formats appearing in soft biomaterial development. In these cases, multiple polymers and treatments can be analyzed by preparing miniaturized chips with thousands of droplets/wells containing material combinations.^[^ ^353^ ^]^ Other approaches facilitate polymer composition and processing parameter screening through a gradient in both parameters either through the use of microfluidics or by variations in material deposition during 3D printing.^[^ ^353^ ^]^ This miniaturized set‐up represents an ability to simultaneously test biological response across a relatively wide design space.

Beyond being able to make a miniaturized design space, HTS is dependent on particular cell‐biomaterial interactions of interest being translated into simple read‐outs. Most commonly fluorescence, absorbance, or luminescence measurements are then simultaneously read for the entire design space.^[^ ^357^ ^]^ While these techniques are to an extent already used in biomedical alloy research current implementation lacks many of the game‐changing features developed for HTS drug discovery, including automation, temporal monitoring, simultaneous marker evaluation, and simpler methods of handling.^[^ ^355^, ^358^ ^]^ As such the scale and speed of validation are not yet comparable. More specifically, fluorescence staining for key cell metabolism targets and cell membrane integrity can be measured and imaged in real time and coupled with automated image recognition software in a plate reader pipeline, offering an array of complementary outputs for HTS cell toxicology.^[^ ^359^ ^]^ In the pharmaceutical sector specific disease markers are also routinely used to facilitate the selection of “hits” that will become prototypes for the next stage of screening. This is facilitated by combining molecules capable of being evaluated by fluorescence polarization (FP), fluorescence correlation spectroscopy (FCS), fluorescence intensity distribution analysis (FIDA), or scintillation proximity assays (SPA), such that real‐time gene transcription, surface receptor activation or cell metabolism can be analyzed.^[^ ^360^ ^]^ Coupling these methods with the production of a miniaturized alloy design space could help to enable HTS of novel compositions and microstructures (Table 1). That said, poor correlations have been observed between conventional 2D in vitro cell culture assays and in vivo biomaterial outcomes.^[^ ^124^ ^]^ As such it is critical that HTS approaches are based on reliable markers, which creates a need for novel models that may enable mechanistic understanding and deliver confidence in using in vitro data to down‐select novel biomedical alloys. The use of spheroids, organoids, or 3D cultures incorporating scaffolds may find utility here.^[^ ^359^ ^]^ While novel rapid bench‐based techniques for the assessment of target biological criteria that would traditionally take weeks of culture (e.g., biomineralization) present another option for alleviating bottlenecks at the validation stage.^[^ ^235^ ^]^

HTS datasets of biological outcomes also present an opportunity to leverage recent advances in machine learning (ML) algorithms.^[^ ^361^ ^]^ Usually, these large material design spaces are probed by brute force with all variations being assessed or refined by technical expertise. Bayesian optimization for the design of experiments is an alternative use of ML models to unearth and implement discovered knowledge throughout batches to suggest future design spaces for analysis.^[^ ^362^ ^]^ As such, further data keeping, analysis, and model development can then be used to facilitate HTS in the early stages of alloy design and reduce the sample number of more advanced or costly experiments. Taken collectively it is clear that embedding of HTS is a critical step to enabling translation of novel biomedical alloys with expanded biological merit indices. While there is inspiration to be taken from the pharmaceutical field and other classes of biomaterials there are still numerous critical modifications needed (e.g., sample preparation) to these methods to ensure they will enable the alloy development community to unpick intricacies surrounding composition and microstructure sensitivities.

## 5 Future Steps and Considerations

While much of the focus has been on creating materials that are safe, biocompatible, and encourage favorable biological responses, it is equally essential to meet the mechanical properties that ensure implant longevity. For example, mechanical properties like wear resistance and fatigue strength are crucial to consider, impacting the long‐term functionality and durability of implants. Corrosion resistance is another essential factor, influencing both mechanical integrity and biocompatibility. Though pure Ti has been extensively evaluated in terms of its biocompatibility and corrosion resistance, the considered alloys and their components should be individually tested.^[^ ^363^, ^364^, ^365^, ^366^ ^]^ Key factors such as alloy composition and microstructure affect corrosion behavior, with methods like electrochemical testing and immersion studies used to assess and optimize resistance.^[^ ^365^, ^367^, ^368^ ^]^ However, the ability to control the surface properties, particularly the passive oxide layer formed on Ti alloy surfaces, is key to influence the corrosion performance.^[^ ^369^, ^370^ ^]^

Surface engineering techniques provide additional opportunities to enhance the performance of biomedical alloys. Coatings can significantly improve corrosion resistance, reduce wear, and promote better cell adhesion, allowing for the customization of implants for specific medical needs.^[^ ^371^, ^372^ ^]^ Furthermore, the material surface plays a crucial role in the response of the biological environment to the biomedical device. Often the ideal surface properties differ from those required by the bulk material. Thus, surface modification techniques may be optimized to retain the excellent bulk attributes of Ti and its alloys, but also improve specific surface properties dictated by the application, for example, improved osseointegration, minimizing bacterial adhesion, or modulating cell differentiation.^[^ ^373^ ^]^

Despite these technological advancements, the commercialization of new biomedical alloys remains a significant challenge, as it aims to balance functionality, safety, scalability, and cost. Gaining regulatory approval requires extensive biological and mechanical testing to meet strict standards, a process that is both time‐consuming and costly. If these new titanium‐based materials are deemed to be bioactive (i.e., they influence cellular metabolism), this positioning them closer to medicinal products and combinatorial devices rather than traditional implants. This introduces a need for rigorous efficacy and safety testing, comprehensive regulatory oversight, and increased financial investment to navigate the development pathway.^[^ ^374^, ^375^ ^]^ Lessons can be taken from the commercialization of bioactive glasses as described by Shearer et al.^[^ ^375^ ^]^ A major challenge is the lack of global alignment in medical device regulations. Addressing this issue is essential to 1) reduce regulatory barriers, 2) facilitate smoother international trade of medical devices, and 3) decrease the regulatory and implementation costs for both governments and industries.^[^ ^376^ ^]^ However, incremental modifications to alloys face considerably fewer regulatory hurdles, which promotes the continued use of similar or identical materials under schemes like the 510(k) pathway in the USA, ultimately limiting innovation.^[^ ^377^ ^]^ Additionally, integrating more exotic elements into Ti alloys (e.g., Ce, Ga, and Ge) raises questions about toxicity, supply chain constraints, and reactivity, as some elements are scarce or difficult to handle. Further considerations on the cost of alloying elements also need to be considered, researchers have begun to focus on low‐cost elements, e.g., Fe, Mn, and Cr as β‐stabilizing elements.^[^ ^301^, ^378^ ^]^ Furthermore, scalability remains a major issue; conventional methods, like casting, are ideal for high production volumes, while additive manufacturing suits smaller, custom batches but introduces challenges like atomization and pre‐alloyed powders, posing significant technical and economic barriers.^[^ ^379^, ^380^ ^]^ Ultimately, the path to commercializing biomedical alloys will require a coordinated effort to streamline regulatory processes and address scalability challenges, supporting the development of safer and more accessible medical devices globally.

## 6 Conclusion

This review emphasizes the need for new Ti‐alloys specifically designed to resist biological threats, whilst meeting the biomechanical performance metrics, of permanent orthopedic implants. By delving into the intricacies of the biological processes governing implant responses, including immune response, angiogenesis, osseointegration, and infection susceptibility, it becomes evident that a greater understanding of how metallic elements influence these mechanisms is essential. This reinforces the need to establish more relevant design criteria, not just for Ti‐alloys but more generally for the biomedical field. Incorporating these insights, and big data, the potential of machine learning could become a pivotal tool to uncover the complex relationships between the alloying elements and biological functionalities. Despite many recent advancements, such as an understanding of macrophage polarization and novel predictive methods, further research is required to combat the scarcity of available data. This necessitates the requirement of widespread adoption and standardization of experimental protocols to facilitate collaboration and data‐sharing across research communities.

To overcome these challenges, the utilization of high‐throughput combinatorial experimental techniques offers a promising avenue, enabling the rapid production of diverse alloy compositions, microstructures, and phase distribution. Various manufacturing methods such as traditional melt, to advanced and cutting‐edge techniques, including powder metallurgy techniques, thin film deposition, and in situ alloy techniques derived from additive methods, have been discussed. Each approach has been scrutinized, presenting its own advantages and limitations, highlighting the need for a diverse array of tools to effectively facilitate biomedical alloy development. Furthermore, the use of machine learning techniques is increasing to help uncover relationships between alloying elements and material properties. Similar to practices demonstrated in other industries, such as the pharmaceutical sector's utilization of modeling to generate compound libraries, these combinatorial techniques aim to enhance the pace of discovery.

To advance the commercialization of biomedical titanium alloys, future efforts must emphasize balancing mechanical integrity with biological compatibility to ensure long‐term implant functionality. Mechanical properties such as wear resistance and fatigue strength remain crucial, while corrosion resistance continues to demand careful alloy selection and testing. Surface engineering techniques, including coatings and surface modifications, offer valuable enhancements in biocompatibility and durability, adapting implants to specific medical applications. Despite progress, regulatory approval, extensive testing, and economic challenges hinder large‐scale adoption. In particular, schemes such as the 510 (k) pathway in the United States inhibit the development of novel innovative medical devices due to the ease and cost benefits of introducing similar products to those already on the market. Future efforts should focus on refining these processes and fostering partnerships with aerospace and medical sectors to meet stringent medical standards, thereby paving the way for safe, scalable, and cost‐effective titanium alloy implants.

This review clearly highlights that embracing interdisciplinary collaboration between material science, biology, and data science, is pivotal to unlocking the full potential of biomedical alloy development. By doing so, researchers can hope to expedite the discovery and validation of novel alloys that offer new functional benefits, which may ultimately enable the creation of safer and more efficacious implants that may transform patient outcomes.

## Conflict of Interest

The authors declare no conflict of interest.
